# Association between hyperglycaemia and adverse perinatal outcomes in south Asian and white British women: analysis of data from the Born in Bradford cohort

**DOI:** 10.1016/S2213-8587(15)00255-7

**Published:** 2015-10

**Authors:** Diane Farrar, Lesley Fairley, Gillian Santorelli, Derek Tuffnell, Trevor A Sheldon, John Wright, Lydia van Overveld, Debbie A Lawlor

**Affiliations:** a Bradford Institute for Health Research, Bradford Teaching Hospitals, Bradford, UK; b Department of Health Sciences, University of York, York, UK; c Hull York Medical School, University of York, York, UK; d Clinical Trials Research Unit, University of Leeds, Leeds, UK; e Bradford Women's and Newborn Unit, Bradford Teaching Hospitals, Bradford, UK; f Radboud University, Nijmegen, Netherlands; g MRC Integrative Epidemiology Unit, University of Bristol, Bristol, UK; h School of Social and Community Medicine, University of Bristol, Bristol, UK

## Abstract

**Background:**

Diagnosis of gestational diabetes predicts risk of infants who are large for gestational age (LGA) and with high adiposity, which in turn aims to predict a future risk of obesity in the offspring. South Asian women have higher risk of gestational diabetes, lower risk of LGA, and on average give birth to infants with greater adiposity than do white European women. Whether the same diagnostic criteria for gestational diabetes should apply to both groups of women is unclear. We aimed to assess the association between maternal glucose and adverse perinatal outcomes to ascertain whether thresholds used to diagnose gestational diabetes should differ between south Asian and white British women. We also aimed to assess whether ethnic origin affected prevalence of gestational diabetes irrespective of criteria used.

**Methods:**

We used data (including results of a 26–28 week gestation oral glucose tolerance test) of women from the Born in Bradford study, a prospective study that recruited women attending the antenatal clinic at the Bradford Royal Infirmary, UK, between 2007 and 2011 and who intended to give birth to their infant in that hospital. We studied the association between fasting and 2 h post-load glucose and three primary outcomes (LGA [defined as birthweight >90th percentile for gestational age], high infant adiposity [sum of skinfolds >90th percentile for gestational age], and caesarean section). We calculated adjusted odds ratios (ORs) and their 95% confidence intervals (CIs) for a 1 SD increase in fasting and post-load glucose. We established fasting and post-load glucose thresholds that equated to an OR of 1·75 for LGA and high infant adiposity in each group of women to identify ethnic-specific criteria for diagnosis of gestational diabetes.

**Findings:**

Of 13 773 pregnancies, 3420 were excluded from analyses. Of 10 353 eligible pregnancies, 4088 women were white British, 5408 were south Asian, and 857 were of other ethnic origin. The adjusted ORs of LGA per 1 SD fasting glucose were 1·22 (95% CI 1·08–1·38) in white British women and 1·43 (1·23–1·67) in south Asian women (p_interaction with ethnicity_ = 0·39). Results for high infant adiposity were 1·35 (1·23–1·49) and 1·35 (1·18–1·54; p_interaction with ethnicity_=0·98), and for caesarean section they were 1·06 (0·97–1·16) and 1·11 (1·02–1·20; p_interaction with ethnicity_=0·47). Associations between post-load glucose and the three primary outcomes were weaker than for fasting glucose. A fasting glucose concentration of 5·4 mmol/L or a 2 h post-load level of 7·5 mmol/L identified white British women with 75% or higher relative risk of LGA or high infant adiposity; in south Asian women, the cutoffs were 5·2 mmol/L or 7·2 mml/L; in the whole cohort, the cutoffs were 5·3 mmol/L or 7·5 mml/L. The prevalence of gestational diabetes in our cohort ranged from 1·2% to 8·7% in white British women and 4% to 24% in south Asian women using six different criteria. Compared with the application of our whole-cohort criteria, use of our ethnic-specific criteria increased the prevalence of gestational diabetes in south Asian women from 17·4% (95% CI 16·4–18·4) to 24·2% (23·1–25·3).

**Interpretation:**

Our data support the use of lower fasting and post-load glucose thresholds to diagnose gestational diabetes in south Asian than white British women. They also suggest that diagnostic criteria for gestational diabetes recommended by UK NICE might underestimate the prevalence of gestational diabetes compared with our criteria or those recommended by the International Association of Diabetes and Pregnancy Study Groups and WHO, especially in south Asian women.

**Funding:**

The National Institute for Health Research.

## Introduction

Gestational diabetes increases the risk of several adverse perinatal outcomes.^1^ In recent years, there has been much debate about how gestational diabetes should be diagnosed. In 2010, the International Association of Diabetes and Pregnancy Study Groups (IADPSG) recommended new thresholds for the diagnosis of the disease, which aimed to reduce obesity risk by identifying infants who were large for gestational age (LGA), with high adiposity at birth, and who had high concentrations of cord-blood C-peptide.^2^ In 2013, WHO, whose previous criteria for diagnosing gestational diabetes have been widely used, endorsed the IADPSG criteria.^3^

**Research in context**

**Evidence before this study**

We searched MEDLINE and MEDLINE in Process, Embase, CINAHL Plus, Cochrane Central Register of Controlled Trials (CENTRAL), Cochrane Database of Systematic Reviews (CDSR), Database of Abstracts of Reviews of Effectiveness (DARE), Health Technology Assessment database (HTA), NHS Economic Evaluation Database (NHS EED), and the Cochrane Methodology Register for various combinations of the terms including: “gestational diabetes” or “diabetes” and “pregnancy” and “birth injury” or “macrosomia” or “large for gestational age” or “labour complications” or “shoulder dystocia” or “fracture” (“clavicle”, “humerus”, “shoulder”, “arm”) or “Erb's palsy”or “pre-eclampsia” or “eclampsia” or “cardiovascular disease” or “obesity” or “hypoglycaemia” or “neonatal unit admission” or “special care unit admission”. In initially screening papers for eligibility, we focused exclusively on those with titles in English (noting that the databases we search provide English titles for most papers irrespective of the language in which the main paper is written). We included papers in any language so long as we were able to obtain a translation. We undertook three searches between March, 2013, and October, 2014, using the same databases and terms, and identified 28 eligible studies (all in English language) investigating the association between gestational glucose concentrations and adverse perinatal outcomes. The appendix shows the full list of references. Most studies were observational, included a general obstetric population, and were undertaken in several countries. Studies used different methods, glucose tolerance testing strategies, and assessed different outcomes. Generally, existing studies showed monotonic associations of maternal glucose concentrations with adverse perinatal outcomes (specifically large for gestational age [LGA], macrosomia, and caesarean section) and no evidence of a threshold effect. There was no strong evidence for an increase in odds of preterm delivery with greater maternal glucose. We did not identify any previous study that compared associations of glucose with perinatal outcomes between south Asian and white European women.

**Added value of this study**

We hypothesised that the association between gestational diabetes and perinatal outcomes, and the criteria for diagnosing gestational diabetes, might differ in south Asian compared with white British women. Our findings show that, as in white British women, women of south Asian origin have graded linear associations of fasting and 2 h post-load glucose with adverse perinatal outcomes including LGA and higher birth adiposity (as defined by skinfold thicknesses >90th percentile). Our data suggest lower thresholds for diagnosing gestational diabetes in south Asian women than white European women.

**Implications of all the available evidence**

In view of the linear association of pregnancy glucose concentrations with adverse perinatal outcomes in south Asian, as in white European women, reducing the thresholds used to diagnose gestational diabetes will identify more women at risk of adverse perinatal outcomes. Effective, safe, and cheap treatments are available for gestational diabetes that reduce glucose concentrations across its range and improve perinatal outcomes. Therefore, use of the criteria we developed in this study, or the International Association of Diabetes and Pregnancy Study Groups (IADPSG)/WHO criteria for diagnosing gestational diabetes in white European women, and our ethnic-specific criteria for south Asian women, might improve perinatal outcomes. However, any effect of lowering the thresholds to diagnose gestational diabetes on long-term offspring health requires assessment in large studies with data from maternal oral glucose tolerance tests and long-term detailed assessment of offspring.

The IADPSG criteria were produced with results from the Hyperglycemia and Adverse Pregnancy Outcomes (HAPO) study,^4^ which aimed to establish the association between maternal glucose concentrations that did not meet criteria for overt diabetes (pre-existing diabetes or gestational diabetes) and risk of adverse perinatal outcomes. HAPO found graded linear associations of fasting and post-load maternal glucose with LGA, high adiposity, and high concentrations of cord-blood C-peptide, and similar linear associations with several other perinatal outcomes. In view of the absence of any clear threshold of glucose concentration at which risk of adverse outcomes increased, the IADPSG reached a consensus on how to calculate the new criteria. They decided that the thresholds for diagnosing gestational diabetes would be: the glucose values at which the odds ratios (ORs) reached 1·75 for birthweight greater than the 90th percentile, percent infant body fat (based on skinfolds) greater than the 90th percentile, and concentration of cord C-peptide greater than the 90th percentile.^2^ Although in most populations the application of the IADPSG criteria increases the number of women diagnosed with gestational diabetes compared with most previously used criteria (table 1),^8^ they might not identify women at risk who have a high 2 h post-load glucose but still below that specified by the IADPSG criteria.^4^

It is unclear whether the association between maternal glucose and perinatal outcomes and the IADPSG criteria for diagnosing gestational diabetes should be the same in south Asian women, who are at higher risk of gestational diabetes than white European women.^9^ The shift in the aim of diagnosing gestational diabetes from one of identifying women at risk of type 2 diabetes to one of identifying risk of future offspring obesity is especially important for south Asians, because south Asian women, on average, have infants of markedly lower birthweight and a reduced risk of LGA than white European women.^7^, ^8^ However, lower birthweight of south Asian infants masks a propensity to greater adiposity and associated cardiometabolic risk in later life.^10^, ^11^, ^12^, ^13^, ^14^, ^15^, ^16^ High maternal pregnancy glucose is an important mediator of greater birth adiposity in south Asian compared with white European infants.^6^ Although findings of the HAPO study showed similar associations across different geographical centres, there were no south Asian centres, and too few south Asian participants to assess the association between maternal glycaemia and perinatal outcomes.

We aimed to establish whether the IADPSG criteria for diagnosis of gestational diabetes are appropriate for south Asian women and to assess how the prevalence of gestational diabetes varies when different criteria for its diagnosis are used in south Asian and white British women. Our specific objectives were to establish the nature of the association of fasting and post-load glucose with adverse perinatal outcomes in a large cohort of south Asian women and compare those findings with a similarly sized cohort of white British women; to use our results to identify appropriate thresholds for diagnosing gestational diabetes in south Asian and white British women; and to compare the prevalence of gestational diabetes in these two groups with different criteria. We hypothesised that the association between fasting and post-load glucose and birthweight and infant adiposity, and the thresholds used to diagnose gestational diabetes, would differ between south Asian and white British women. Furthermore, we predicted that prevalence of gestational diabetes would be greater in south Asian women than white British women irrespective of criteria used. Our findings should inform clinical practice for diagnosing gestational diabetes.

## Methods

### Study design and participants

**Figure 1:**
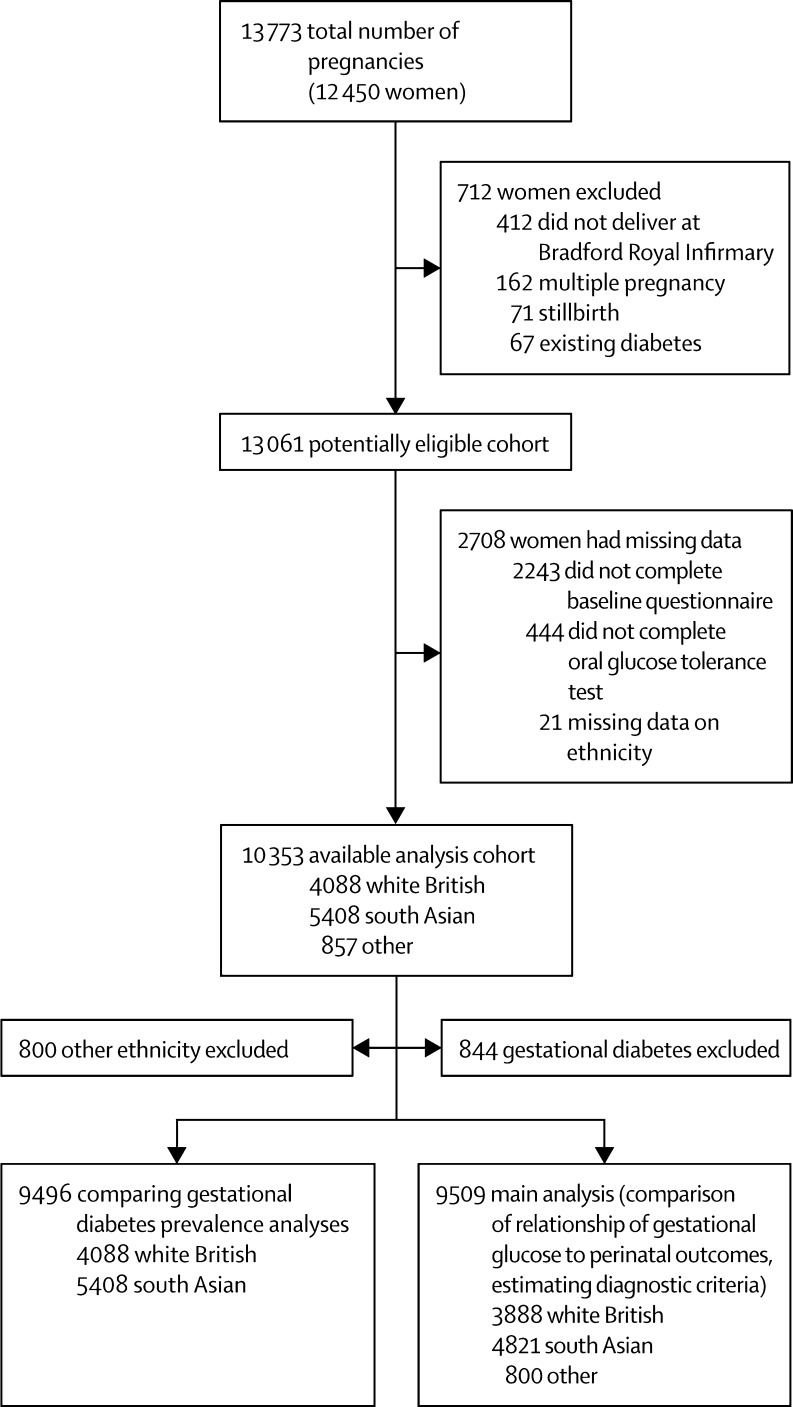
Study sample The criteria used in the hospital in which study participants were recruited to diagnose gestational diabetes (and hence exclude them from the analyses presented here) were either fasting glucose ≥6·1 mmol/L or 2 h post-load glucose ≥7·8 mmol/L.

Born in Bradford^17^ is a prospective birth cohort study of women who delivered a live singleton baby at the Bradford Royal Infirmary, Bradford, UK. Figure 1 shows full inclusion and exclusion of women from Born in Bradford in this study. Women were excluded from all analyses if they did not complete a baseline questionnaire or the oral glucose tolerance test or had missing data for ethnic origin. For the main analyses of the association of gestational glucose with perinatal outcomes and development of gestational diabetes diagnostic criteria, we excluded women diagnosed with gestational diabetes. Gestational diabetes was defined according to modified WHO criteria operating at the time (either fasting glucose ≥6·1 mmol/L, or 2 h post-load glucose ≥7·8 mmol/L).^5^, ^6^

The cohort is broadly representative of the obstetric population in Bradford.^17^ All women booked for delivery in Bradford are offered a 75 g oral glucose tolerance test (comprising fasting and 2 h post-load samples) at around 26–28 weeks' gestation, and women were recruited mainly at their oral glucose tolerance test appointment. At recruitment, women had their height and weight measured, completed an interviewer-administered questionnaire, and provided written consent for information to be abstracted from their medical records. Interviews were done in English or in south Asian languages (including Urdu and Mirpuri). Analysis of glucose samples was done using a Siemens Advia 2400 analyser following a standard protocol. The coefficients of variation range between 1·73% at 3·2 mmol/L and 0·64% at 19·1 mmol/L. Ethics approval was obtained from the Bradford Research Ethics Committee (07/H1302/112). All participants provided informed written consent.

### Outcomes

We assessed associations of maternal glucose concentrations with three primary outcomes: LGA (defined as birthweight greater than the 90th percentile for gestational age), infant adiposity (defined as sum of skinfolds greater than the 90th percentile for gestational age), and caesarean section; and five secondary outcomes: pre-eclampsia, preterm delivery, shoulder dystocia, instrumental vaginal delivery, and admission to the neonatal unit. These outcomes are established clinical complications of gestational diabetes, and similar to the primary and secondary outcomes in the HAPO study. We did not have information about cord-blood C-peptide or neonatal hypoglycaemia in our cohort. We were unable to calculate percentage body fat from skinfolds as done in HAPO because no equivalent formulae exist for south Asian infants; thus, we used a cutoff of greater than the 90th percentile for the sum of skinfolds. We included caesarean section in our analyses because although it is not used to predict future risk of adiposity and ill health, it is an important perinatal outcome and is associated with LGA, greater infant adiposity, and increased health service costs.^18^

Birthweight, mode of delivery (normal vaginal, instrumented vaginal, or caesarean section), gestational age, pre-eclampsia, shoulder dystocia, and admission to the neonatal unit were obtained from hospital records. Caesarean section was compared with all vaginal deliveries. Pre-eclampsia was defined as new-onset proteinuria (>300 g in 24 h) together with blood pressure of 140/90 mm Hg or higher after 20 weeks' gestation on more than one occasion. Birthweights were converted into standard deviation scores standardised for gestational age and sex relative to the UK-WHO growth standard.^19^, ^20^ Infants were then categorised as either being greater than the 90th percentile or not.^11^ The UK-WHO growth standards are based on data from six counties (USA, Norway, Oman, Brazil, India, and Ghana) and describe the optimum pattern of growth for all children, rather than the prevailing pattern in the UK.^20^ Skinfold thickness (triceps and subscapular) were summed and the 90th percentile was established from quantile regression using six sex–ethnic groups (combining sex, and ethnic origin [white British, south Asian, and other]) and adjusted for parity (0, 1, 2, 3+).^21^ The intra-rate and inter-rate technical error of measurements for the skinfold thicknesses were, respectively, 0·22 to 0·35 mm and 0·15 to 0·54 mm for triceps, and 0·14 to 0·25 mm and 0·17 to 0·63 mm for subscapular skinfolds.^22^

### Statistical analysis

Associations of fasting and post-load glucose with outcomes were assessed by categories, and with glucose as a continuous variable (per SD). We used multivariable logistic regression with clustered sandwich estimators^23^ (to account for some women in the cohort having more than one pregnancy) to assess associations of fasting and post-load glucose with each outcome. We followed the analytical protocol used in the HAPO study as closely as possible, with fasting and post-load glucose concentrations divided into seven categories (see table 2 footnotes for definition of categories). In order to explore any extreme threshold effects, the top two categories for fasting and post-load glucose included about 1% and 3% of women, respectively.

Models were adjusted for gestational age at oral glucose tolerance test, presence or absence of family history of diabetes, family history of hypertension, previous gestational diabetes, previous macrosomia, smoking status, alcohol consumption during pregnancy, maternal age and BMI, maternal education, baby sex, and parity. Models for all women were additionally adjusted for ethnic origin. Models for south Asian women were not adjusted for alcohol consumption during pregnancy because most reported never drinking alcohol. Additionally, preterm delivery was adjusted for squared maternal BMI because of evidence of a quadratic relationship of BMI with preterm delivery. Shoulder dystocia models were not adjusted for previous gestational diabetes due to small numbers. The appendix provides full details of the categorisation of these variables.

We established fasting and post-load glucose thresholds for birthweight greater than the 90th percentile and standardised sum of skinfolds greater than the 90th percentile that equated to an odds ratio (OR) of 1·75, using the methods of the IADPSG^2^ (appendix).

All analyses were undertaken separately in white British and south Asian women and we tested for differences in associations by including an interaction term between glucose and ethnic origin. Because women of south Asian origin were mainly Pakistani, we undertook a sensitivity analysis in which we repeated analyses only including Pakistani women.

To maximise statistical power and minimise bias that might occur if women with missing data were excluded from analyses, we used multivariate multiple imputation with chained equations to impute missing values^24^ (appendix). We repeated all analyses with the complete data cohort for comparison. The appendix gives additional details of the statistical analyses.

### Role of the funding source

The funding bodies had no role in study design, data collection or analysis, decision to publish, or preparation of the manuscript. The corresponding author had access to all data and made the final decision to submit for publication.

## Results

Women were recruited to the Born in Bradford study between March, 2007, and November, 2010; investigators collected detailed information about 12 450 women (13 773 pregnancies resulting in 13 818 births). After exclusions, 9509 women (4821 south Asian and 3888 white British) were included in the main analyses looking at associations of fasting and post-load glucose with adverse perinatal outcomes. 844 women with gestational diabetes excluded from main analyses were included in the analyses that compared the prevalence of gestational diabetes with different criteria. The appendix shows characteristics of the women and infants in the eligible cohort: 51% were south Asian, 41% were white British, and 8% were of other ethnic origin. Median fasting and post-load glucose concentrations were slightly higher in south Asian than white British women. White British infants were almost three times more likely than south Asian infants to have a birthweight greater than the 90th percentile, but the frequency of sum of skinfolds greater than the 90th percentile was similar in white British and south Asian infants. Characteristics were similar in the larger cohort of eligible women as in those included in the main analysis cohort (appendix).

**Figure 2:**
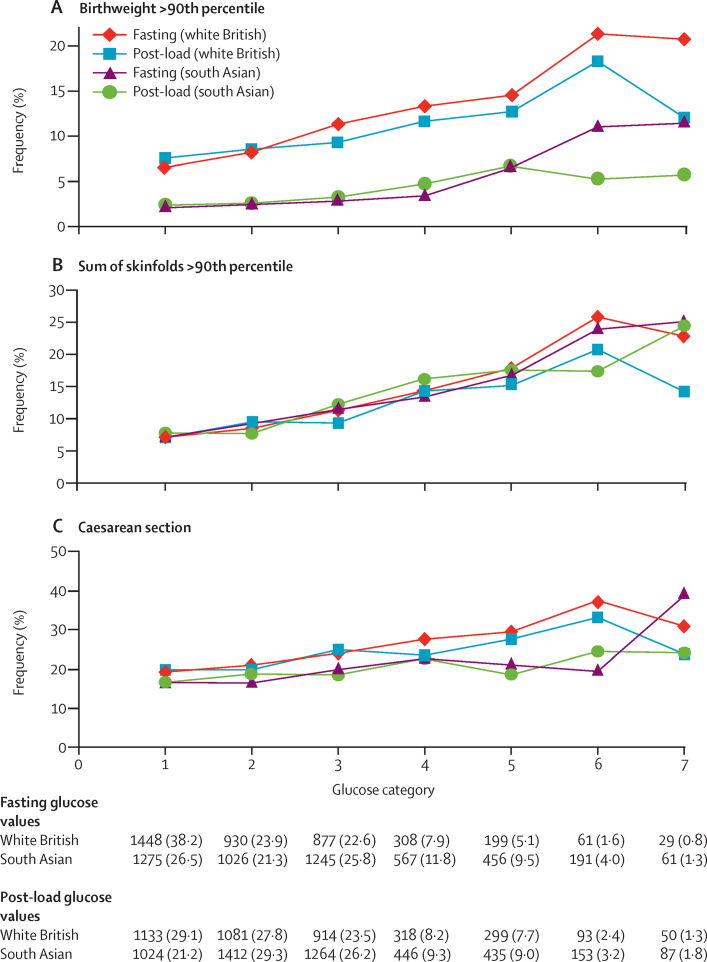
Frequency of primary outcomes across glucose categories by ethnic origin (white British [N=3888] and south Asian [N=4821]) Glucose categories are defined as follows: fasting plasma glucose level category 1: <4·3 mmol/L; category 2: 4·3–4·4 mmol/L; category 3: 4·5–4·7 mmol/L; category 4: 4·8–4·9 mmol/L; category 5: 5·0–5·2 mmol/L; category 6: 5·3–5·6 mmol/L; category 7: 5·7–6·0 mmol/L. Post-load plasma glucose level category 1: <4·7 mmol/L; category 2: 4·7–5·4 mmol/L; category 3: 5·5–6·2 mmol/L; category 4: 6·3–6·6 mmol/L; category 5: 6·7–7·2 mmol/L; category 6: 7·3–7·5 mmol/L; category 7: 7·6–7·7 mmol/L.

Figure 2 shows the unadjusted percentage of women in each group that had each of the three primary outcomes by categories of fasting and 2 h post-load glucose. Generally, the frequency of each of the three primary outcomes increased across the seven categories of fasting and post-load glucose, with no evidence of a threshold at which risk markedly increases, except for the association of fasting glucose with caesarean section in south Asian women. The higher prevalence of birthweight greater than the 90th percentile in white British infants, compared with south Asian, is consistent across all glucose categories. Combining data for all women (ie, including 99% of the cohort) showed monotonic relationships of fasting and post-load glucose up to the 6th category (appendix).

Regression analyses confirmed monotonic associations of glucose with each of the primary outcomes, in each group, without (appendix) and with adjustment for confounders (table 2). In view of the monotonic nature of the associations, we focused our comparisons on results with fasting or post-load glucose as a continuous variable (per 1 SD). Although there was not strong statistical evidence of differences, the point estimates suggested stronger associations of fasting and post-load glucose with all three outcomes except for those of fasting glucose with LGA and post-load glucose with Caesarean section. However, there was no strong statistical evidence that the associations differed between the two groups for any primary outcome (p_interaction_ ≥0·2 for all associations).

Associations with secondary outcomes were similar in the two ethnic groups (appendix). The frequency of pre-eclampsia, shoulder dystocia and, with a weaker magnitude, instrumental delivery, also increased across each glucose category, especially with fasting glucose (appendix). Neither fasting nor post-load glucose concentrations were clearly associated with preterm delivery or admission to the neonatal unit.

Table 3 shows the thresholds of fasting and 2 h post-load glucose that would result in an OR of 1·75 for birthweight greater than the 90th percentile, and sum of skinfolds greater than the 90th percentile in each group. Fasting and post-load glucose thresholds based on the average of birthweight and skinfolds greater than the 90th percentile for all women irrespective of ethnic origin were 5·3 mmol/L and 7·5 mmol/L, respectively. Fasting glucose thresholds based on birthweight or the average of birthweight and skinfolds greater than the 90th percentile were higher for white British than south Asian women (table 3); with skinfolds greater than the 90th percentile alone as the outcome, the fasting glucose threshold was the same in both ethnic groups. There was no 2 h post-load threshold that reached an OR of 1·75 for birthweight greater than the 90th percentile in either ethnic group. A threshold for sum of skinfolds greater than the 90th percentile was only found in south Asian women (table 3).

Table 4 shows gestational diabetes prevalence by past and present diagnostic criteria, and the criteria derived from our data. For our study criteria, we show prevalences with the same thresholds in both ethnic groups (the thresholds derived for all women) and also ethnic-specific thresholds. Prevalence of gestational diabetes was about twice as high in south Asian women using any criteria (range 4·1–17·4%) than in white British women (1·2–8·7%) for all (non-ethnic specific) criteria. Prevalence was greater in both ethnic groups with the recently derived IADPSG, UK NICE, and our criteria compared with the 1999 WHO criteria. Of the three recent criteria, the UK NICE criteria resulted in the lowest prevalences in white British women and our criteria the highest. In south Asian women, the UK NICE criteria resulted in the lowest prevalence. If we applied criteria derived in our study for all women (ie, not taking account of ethnic origin) to the south Asian women, the prevalence of gestational diabetes was the same using either IADPSG/WHO or our criteria. However, when we applied our ethnic-specific criteria, prevalence in south Asian women was nearly three times that in white British women (table 4).

The amount of missing data ranged from 0 to 32% for the different variables (appendix) and 5056 of the 9509 (53%) had complete data on all variables for the main analyses. Distributions of any variable with missing data were the same in the imputation datasets and for observed complete case data (appendix). Regression analyses using only participants with complete data gave similar results to those undertaken on the multiple imputed datasets presented here (appendix). There was no strong evidence for a quadratic curvilinear association between fasting or post-load glucose and any of the primary or secondary outcomes (appendix). Results of analyses restricted to Pakistani women did not differ from those presented for all south Asian women (appendix).

## Discussion

We recorded graded monotonic associations of fasting and 2 h post-load glucose with LGA and high adiposity (as assessed by skinfold thickness) across most of the glucose distribution in both south Asian and white British women. The associations of glucose with LGA appeared stronger in south Asian than white British women, but there was no statistical evidence of an interaction with ethnic origin. Applying the same method as the IADPSG to our data, we estimated fasting and post-load glucose thresholds for diagnosing gestational diabetes that are lower in south Asian compared with white British women. For white British women, our criteria included a fasting glucose threshold that was slightly higher, and a 2 h post-load glucose threshold that was markedly lower, than those recommended by the IADPSG and WHO. Our results support a lower threshold for both fasting and post-load glucose for diagnosing gestational diabetes than is currently recommended by the UK NICE in both white British and south Asian women. The UK NICE supports higher fasting glucose thresholds to those proposed by the IADPSG and WHO in white British and south Asian women, but lower 2 h post-load glucose thresholds. Using existing criteria, the prevalence of gestational diabetes in our cohort was about twice as high in south Asian than in white British women; when we applied the ethnic-specific criteria derived from our data, the prevalence was three times higher in south Asian women, and identified about 25% of south Asian women as having gestational diabetes.

Overall patterns of associations in our study, for both primary and secondary outcomes, were similar to those seen in the HAPO study, especially for fasting glucose.^4^ Because of differences between ours and the HAPO study in the post-load glucose threshold used to exclude women from the study cohort, our highest 2 h post-load category (category 7) was similar to category 4 in the HAPO study. As a result, for some outcomes, the linear relationship seems to flatten at the upper end of the 2 h post-load glucose categories.

Compared with the IADPSG, who used data from the HAPO study, we could not identify a 2 h post-load threshold: there was no threshold that reached an OR of 1·75 for birthweight greater than the 90th percentile, and only south Asian women reached a threshold for this OR for sum of skinfolds greater than the 90th percentile. The IADPSG consensus panel chose 1·75 to represent the lowest level of clinically important risk; a lower OR was not considered clinically important. Gestational diabetes was diagnosed in our study using a lower 2 h post-load glucose threshold than in the HAPO study; both studies excluded women with gestational diabetes as it would be unethical not to treat them. If we had applied the same high 2 h post-load glucose threshold as in HAPO to diagnose gestational diabetes and to exclude women from the main analysis, we would have been more likely to identify an OR of 1·75, because women with higher glucose concentrations and greater associated risk of the primary outcomes would have been included in our analyses. The 2 h post-load glucose used to exclude women with gestational diabetes in HAPO was much higher than that recommended by WHO, and also by other criteria recommended at the time that the HAPO study began, including the Australasian Diabetes in Pregnancy Society criteria. Thus, the 2 h post-load glucose threshold used to define gestational diabetes in the IADPSG and WHO criteria is higher than that suggested by our study (table 1). Because the diagnostic criteria for gestational diabetes in our study meant that we excluded women from the main analyses with a much lower post-load glucose threshold than was the case in the HAPO study, we had difficulty identifying a glucose threshold that reached an OR of 1·75 for sum of skinfolds greater than the 90th percentile in white British women. Therefore, our gestational diabetes diagnostic criteria for this group are mainly driven by results of the associations with LGA.

Consistent with other studies,^25^, ^26^, ^27^, ^28^ we have shown that using any criteria the prevalence of gestational diabetes is greater in south Asian compared with white British women. When we used the same criteria for both ethnic groups, the criteria derived from our study resulted in a higher prevalence of gestational diabetes than the UK NICE criteria for both white British and south Asian women, but broadly similar prevalences for both groups to those found with the IADPSG/WHO criteria. When we used ethnic-group-specific criteria, for white British women prevalences remained higher than UK NICE, but similar to IADPSG/WHO criteria, whilst those for south Asians became higher for both of these other two criteria.

Our study cohort is large and well characterised. The broad consistency of our findings with the results of the HAPO study, and the fact that our results were unchanged when we limited the analyses in south Asians to those of Pakistani origin, suggest the results might be generalisable to all white Europeans and south Asians. Some participants had missing data for some variables, but the distribution of recorded variables and those from the pooled multiple imputed datasets were similar, as were the association results. We did not collect data for 1 h post-load glucose concentrations, which were measured in the HAPO study, and a 1 h post-load glucose threshold is included in IADPSG/WHO criteria for gestational diabetes. Although the HAPO study found linear associations of 1 h glucose with adverse perinatal outcomes, none of the randomised trials that have shown the effect of treatments on adverse perinatal outcomes have used this to define gestational diabetes. Furthermore, it is unclear how many additional women this additional glucose measurement identifies in different populations. Thus, the benefit of this additional measurement remains somewhat unclear. We do not have data for cord-blood C-peptide concentrations or neonatal hypoglycaemia. High cord-blood C-peptide concentrations were one of the criteria used by the IADPSG in the development of their diagnostic criteria; this additional information might have affected our results. However, the similar prevalences of gestational diabetes in white British women using the IADPSG/WHO criteria or our study criteria suggest including these data would not have markedly changed our results.

Concerns have been raised about the increased prevalence of gestational diabetes and hence the cost to health services if the IADPSG criteria are used worldwide in place of the previously widely used 1999 WHO criteria.^9^, ^29^, ^30^ Until the late 1990s, the main aim of diagnosing gestational diabetes was to identify women at risk of subsequent type 2 diabetes.^31^ By contrast, the outcomes used to develop the IADPSG criteria, which we also used, were chosen to identify offspring at risk of future high adiposity and cardiometabolic risk.^31^ Although there is evidence that gestational diabetes causes greater adiposity in offspring in later life,^31^, ^32^ there is still debate about the validity of that evidence.^33^ Thus, the extent to which the IADPSG or our criteria will accurately predict future adverse offspring health remains to be established. Conversely, in view of the graded association of maternal glucose concentrations with adverse perinatal outcomes, lowering the thresholds used to diagnose gestational diabetes would identify more pregnancies at risk of these outcomes. Because effective, safe, and cheap treatments are available for gestational diabetes (eg, lifestyle advice, metformin, and insulin) that reduce glucose across its distribution and help prevent adverse perinatal outcomes,^34^, ^35^ applying the IADPSG/WHO 2013 or our criteria in place of the WHO 1999 criteria, and also in place of the recently suggested UK NICE criteria, might improve perinatal outcomes. Because the UK NICE 2015 criteria recommend higher thresholds of fasting and post-load glucose than the IADPSG/WHO or our newly defined criteria, their use will identify fewer women who are at increased risk of adverse outcomes.^36^

To conclude, our data support the use of lower fasting and post-load glucose thresholds in south Asian than in white British women. They also suggest that compared with our criteria or those of the IADPSG/WHO, the criteria recommended by UK NICE might underestimate the prevalence of gestational diabetes, especially in south Asian women. The use of our ethnic-specific thresholds for diagnosing gestational diabetes in south Asian women, and of either our, or the IASPSG/WHO, criteria for white European women might reduce the occurrence of adverse perinatal outcomes, in particular LGA, as more at-risk women would be treated. However, the cost-effectiveness of applying our criteria, and the effect of applying any of the recently proposed criteria on later-life adiposity and associated cardiometabolic health in offspring are unknown and require further investigation.

## Figures and Tables

**Table 1:** Different criteria used for diagnosis of gestational diabetes

	Glucose thresholds (mmol/L)*			**Years used**	**Coverage of use**
	Fasting	1 h post-load	2 h post-load		
HAPO exclusion^5^	5·8	..	11·1	2002	Some US cities
WHO (previous)^5^	7·0	..	7·8	1999–2013	Widespread worldwide
WHO (previous, modified)^6^	6·1	..	7·8	1999 to present	UK
UK NICE^7^	5·6	..	7·8	2015	UK
IADPSG and WHO (present)^2^, ^3^	5·1	10·8	8·5	2010/13 to present	Widespread worldwide

HAPO=Hyperglycaemia and Adverse Pregnancy Outcomes study. NICE=National Institute for health and Care Excellence. IADPSG=International Association of Diabetes and Pregnancy Study Groups.

* All values are for a glucose tolerance test undertaken at about 26–28 weeks of gestation.

**Table 2:** Confounder-adjusted association of gestational fasting and 2 h post-load glucose with primary outcomes

		**All women (N=9509) OR (95% CI)**	**White British (N=3888) OR (95% CI)**	South Asian (N=4821) OR (95% CI)*	**p**_interaction_*
**Outcome by fasting glucose category**†**and per 1 SD**					
Birthweight >90th percentile					
	1 (Reference)	1·00	1·00	1·00	0·39
	2	1·18 (0·90–1·54)	1·15 (0·83–1·59)	1·07 (0·59–1·94)	..
	3	1·35 (1·04–1·74)	1·38 (1·01–1·90)	1·10 (0·65–1·88)	..
	4	1·42 (1·02–1·97)	1·57 (1·04–2·37)	1·05 (0·56–1·98)	..
	5	1·90 (1·35–2·67)	1·59 (0·97–2·62)	2·12 (1·20–3·76)	..
	6	3·10 (2·00–4·79)	2·21 (1·07–4·54)	3·35 (1·72–6·51)	..
	7	2·60 (1·35–5·04)	2·09 (0·80–5·48)	3·25 (1·29–8·21)	..
	Per 1 SD	1·31 (1·20–1·43)	1·22 (1·08–1·38)	1·43 (1·23–1·67)	..
Sum of skinfolds >90th percentile					
	1 (reference)	1·00	1·00	1·00	0·98
	2	1·11 (0·88–1·40)	1·04 (0·74–1·46)	1·29 (0·92–1·82)	..
	3	1·40 (1·14–1·72)	1·35 (0·96–1·88)	1·56 (1·15–2·13)	..
	4	1·61 (1·24–2·09)	1·69 (1·09–2·62)	1·70 (1·18–2·45)	..
	5	2·02 (1·54–2·64)	2·05 (1·26–3·36)	2·15 (1·49–3·10)	..
	6	3·23 (2·29–4·56)	3·20 (1·52–6·74)	3·18 (2·01–5·02)	..
	7	2·73 (1·53–4·87)	2·71 (0·97–7·58)	3·06 (1·44–6·51)	..
	Per 1 SD	1·35 (1·25–1·45)	1·35 (1·18–1·54)	1·35 (1·23–1·49)	..
Caesarean delivery					
	1 (reference)	1·00	1·00	1·00	0·47
	2	0·98 (0·84–1·13)	1·03 (0·83–1·27)	0·99 (0·79–1·24)	..
	3	1·11 (0·96–1·28)	1·06 (0·86–1·32)	1·20 (0·97–1·49)	..
	4	1·17 (0·97–1·41)	1·11 (0·81–1·51)	1·33 (1·03–1·73)	..
	5	1·20 (0·98–1·48)	1·18 (0·83–1·69)	1·18 (0·88–1·56)	..
	6	1·14 (0·84–1·55)	1·42 (0·83–2·45)	1·02 (0·67–1·56)	..
	7	2·14 (1·34–3·41)	1·25 (0·57–2·77)	2.88 (1·58–5·25)	..
	Per 1 SD	1·09 (1·03–1·15)	1·06 (0·97–1·16)	1·11 (1·02–1·20)	..
Outcome by 2 h post-load glucose category*and per 1 SD					
Birthweight >90th percentile					
	1 (reference)	1·00	1·00	1·00	0·60
	2	0·95 (0·74–1·23)	1·00 (0·73–1·37)	0·96 (0·56–1·66)	..
	3	1·08 (0·83–1·39)	0·98 (0·71–1·36)	1·04 (0·61–1·76)	..
	4	1·29 (0·92–1·80)	1·20 (0·78–1·84)	1·39 (0·72–2·66)	..
	5	1·58 (1·14–2·19)	1·18 (0·76–1·82)	2·12 (1·15–3·93)	..
	6	1·71 (1·04–2·81)	1·74 (0·90–3·36)	1·66 (0·69–3·98)	..
	7	1·29 (0·65–2·60)	1·27 (0·50–3·26)	1·64 (0·54–5·05)	..
	Per 1 SD	1·17 (1·07–1·29)	1·10 (0·98–1·24)	1·28 (1·06–1·55)	..
Sum of skinfolds >90th percentile					
	1 (reference)	1·00	1·00	1·00	0·23
	2	1·02 (0·81–1·29)	1·24 (0·88–1·73)	0·96 (0·68–1·35)	..
	3	1·32 (1·05–1·65)	1·13 (0·78–1·63)	1·51 (1·10–2·07)	..
	4	1·84 (1·40–2·41)	1·76 (1·12–2·76)	1·94 (1·33–2·83)	..
	5	1·94 (1·47–2·55)	1·79 (1·13–2·82)	2·22 (1·52–3·25)	..
	6	2·29 (1·54–3·39)	2·63 (1·35–5·14)	2·13 (1·25–3·64)	..
	7	2·53 (1·53–4·17)	1·80 (0·68–4·77)	3·13 (1·71–5·74)	..
	Per 1 SD	1·31 (1·21–1·42)	1·26 (1·11–1·42)	1·38 (1·23–1·54)	..
Caesarean delivery					
	1 (reference)	1·00	1·00	1·00	0·54
	2	0·95 (0·82–1·10)	0·89 (0·72–1·11)	1·06 (0·84–1·32)	..
	3	1·07 (0·92–1·24)	1·09 (0·87–1·37)	1·01 (0·80–1·27)	..
	4	1·11 (0·91–1·36)	0·96 (0·70–1·32)	1·19 (0·89–1·60)	..
	5	1·00 (0·81–1·23)	1·03 (0·76–1·42)	0·97 (0·71–1·33)	..
	6	1·31 (0·96–1·79)	1·12 (0·68–1·85)	1·35 (0·88–2·07)	..
	7	1·15 (0·76–1·74)	0·86 (0·43–1·72)	1·29 (0·72–2·29)	..
	Per 1 SD	1·05 (0·99–1·11)	1·02 (0·94–1·10)	1·05 (0·96–1·14)	..

* Testing the null hypothesis that the associations of glucose categories with outcome do not differ between white British and south Asian women.

† Glucose categories are defined as follows: fasting plasma glucose level category 1: <4·3 mmol/L; category 2: 4·3–4·4 mmol/L; category 3: 4·5–4·7 mmol/L; category 4: 4·8–4·9 mmol/L; category 5: 5·0–5·2 mmol/L; category 6: 5·3–5·6 mmol/L; category 7: 5·7–6·0 mmol/L. Post-load plasma glucose level category 1: <4·7 mmol/L; category 2: 4·7–5·4 mmol/L; category 3: 5·5–6·2 mmol/L; category 4: 6·3–6·6 mmol/L; category 5: 6·7–7·2 mmol/L; category 6: 7·3–7·5 mmol/L; category 7: 7·6–7·7 mmol/L.

**Table 3:** Thresholds of fasting and post-load glucose concentrations that would identify an odds ratio of roughly 1·75 for birthweight >90th percentile and sum of skinfolds >90th percentile

	**All women (N=10 356)**		**White British women (N=4105)**		**South Asian women (N=5445)**	
	Fasting glucose threshold (mmol/L)	2 h post-load glucose threshold (mmol/L)	Fasting glucose threshold (mmol/L)	2 h post-load glucose threshold (mmol/L)	Fasting glucose threshold (mmol/L)	2 h post-load glucose threshold (mmol/L)
Birthweight >90th percentile	5·3	NP	5·6	NP	5·1	NP
Sum skinfolds >90th percentile	5·2	7·5	5·2	NP	5·2	7·2
Average glucose concentration for both birthweight and sum of skinfolds >90th percentile	5·3	7·5	5·4	NP	5·2	7·2

NP=not possible to work out a threshold because within our study none of the women reached a threshold that gave an odds ratio of 1·75 or greater (the International Association of Diabetes and Pregnancy Study Groups consensus minimal odds ratio deemed to be of clinical importance).

**Table 4:** Prevalence of gestational diabetes in south Asian and white British women using different criteria

	**Criteria (all define gestational diabetes as glucose concentrations at or above one or more of the following)**			**Prevalence in our study population (%, 95% CI)**	
	Fasting glucose (mmol/L)	1 h post-load glucose (mmol/L)	2 h post-load glucose (mmol/L)	White British	South Asian
**Older criteria used**					
Exclusion in HAPO*	5·8	..	11·1	1% (0·9–1·5)	4% (3·6–4·7)
WHO (previous)†	7·0	..	7·8	5% (4·1–5·4)	10% (9·6–11·2)
WHO (previous, modified)‡	6·1	..	7·8	5% (4·3–5·6)	11% (10·0–11·7)
**Recently proposed criteria**					
UK NICE§	5·6	..	7·8	6% (5·2–6·6)	13% (11·7–13·4)
IADPSG/WHO (current)¶	5·1	10·8	8·5	8% (6·8–8·5)	17% (16·3–18·3)
**Our study**					
Same criteria for all women‖	5·3	..	7·5	9% (7·9–9·6)	17% (16·4–18·4)
For white British	5·4	..	7·5	8% (7·5–9·2)	..
For south Asian	5·2	..	7·2	..	24% (23·1–25·3)

HAPO=Hyperglycaemia and Adverse Pregnancy Outcomes study. NICE=National Institute for health and Care Excellence. IADPSG=International Association of Diabetes and Pregnancy Study Groups.

* Used in the HAPO study to exclude women with gestational diabetes.

† Used by WHO up to 2013.^17^

‡ Criteria used for all pregnant women in Bradford (and in other populations) at the time that women were recruited for the Born in Bradford study and used here to exclude those with gestational diabetes.

§ Criteria in current UK guidelines.^36^

¶ Criteria were developed using HAPO data and were adopted by WHO in 2013.^2^, ^3^

‖ Criteria developed in this study.
