# Supplementary material for: Association between hyperglycaemia and adverse perinatal outcomes in south Asian and white British women: analysis of data from the Born in Bradford cohort
Source: Lancet Diabetes Endocrinol. 2015 Oct;3(10):795–804. doi: 10.1016/S2213-8587(15)00255-7 (PMC4673084; doi:10.1016/S2213-8587(15)00255-7)
Supplement: Supplementary appendix [file mmc1.pdf]

THE LANCET Diabetes & Endocrinology

Supplementary appendix

This appendix formed part of the original submission and has been peer reviewed. We post it as supplied by the authors.

Supplement to: Farrar D, Fairley L, Santorelli G, et al. Association between hyperglycaemia and adverse perinatal outcomes in south Asian and white British women: analysis of data from the Born in Bradford cohort. *Lancet Diabetes Endocrinol* 2015; published online Sept 7. http://dx.doi.org/10.1016/S2213-8587(15)00255-7.

On-line Supplementary appendix for:

Association between hyperglycaemia and adverse perinatal outcomes in south Asian and white British women: analysis of data from the Born in Bradford cohort

Diane Farrar,* (PhD)[1, 2] Lesley Fairley, (MSc)[1] Gillian Santorelli, (MSc)[3] Derek Tuffnell, (FRCOG)[4] Trevor A Sheldon, (DSc)[5] John Wright, (FRCP)[1] Lydia van Overveld (MSc)[6] and Debbie A Lawlor (PhD).[7, 8]

[1]Bradford Institute for Health Research, Bradford Teaching Hospitals, Bradford, UK

[2] Department of Health Sciences, University of York, York, UK

[3]Clinical Trials Research Unit, University of Leeds, Leeds, UK

[4]Bradford Women's and Newborn Unit, Bradford Teaching Hospitals, Bradford, UK

[5]Hull York Medical School, University of York, York, UK

[6]Radboud University, Nijmegen, The Netherlands

[7]MRC Integrative Epidemiology Unit at the University of Bristol, Oakfield House, Oakfield Grove, Bristol BS8 2BN, UK

[8]School of Social and Community Medicine, University of Bristol, Bristol, UK

Assessment of covariables

All data on potential confounders were collected from the baseline questionnaire or extracted from the hospital records. The woman's ethnicity, age, education, parity, smoking, and alcohol consumption were obtained from the interviewer administered questionnaire. Ethnicity was categorised as white British, south Asian, or other ethnicity according to UK Office of National Statistics criteria.[1] Education was equivalised to UK standard attainments and participants were included in one of five mutually exclusive categories (<5 GCSE equivalent, +5 GCSE equivalent, A level equivalent, higher, other).[2] Parity was categorised as 0 or $\geq$1 previous pregnancies. Smoking was categorised as never, past (not during this index pregnancy), current (during this pregnancy) and alcohol as consumed during this pregnancy, or not. Because the vast majority of women of south Asian origin (>97%) reported never drinking alcohol, there was insufficient variation in this variable to adjust for it in analyses conducted on south Asian women alone. Given this, alcohol could not confound associations in that group.

Maternal body mass index (BMI) was calculated from height measured at the time of recruitment and from weight measured at booking antenatal clinic, which was obtained from electronic hospital records. Expected date of birth (40 weeks) was estimated from a ~10 week gestation ultrasound scan. Using that date, together with the date of OGTT and date of birth, gestational age at OGTT and birth were calculated. Infant sex was obtained from electronic hospital records and family history of diabetes and hypertension were abstracted from paper hospital records.

Additional statistical analysis details

General approach

We plotted the unadjusted percentage of each outcome across the fasting and post-load categories so that we could visually inspect the nature of the associations, including any evidence of a threshold effect in extreme categories. Multivariable logistic regression with clustered sandwich estimators[3] (to account for multiple pregnancies in some women) was used to examine associations of fasting and 2 hour post-load glucose with each outcome. Associations of fasting and post-load glucose with outcomes were examined by categories and with glucose as a continuous variable. The continuous glucose variables were standardised to enable odds ratios to be calculated for a 1 standard deviation (SD) increase in fasting and post-load glucose levels. To further explore non-linear (curvilinear) associations, a squared term of the continuous glucose values was added to the models for each pregnancy outcome.

We adjusted for potential confounding by gestational age at oral glucose tolerance test (OGTT), maternal age, body mass index (BMI), smoking status, any alcohol during pregnancy, maternal education, family history of diabetes, family history of hypertension, previous gestational diabetes (GDM), previous macrosomic baby, infant sex, and parity. Squared terms for maternal age and BMI were pre-screened for significance in models for each pregnancy outcome that did not include glucose or other covariables; where significant, they were included in the fully adjusted models.

Analyses for determining glucose thresholds that equated to an odds ratio of 1·75

We determined fasting and 2 h post-load thresholds for birthweight >90th percentile and standardised sum of skinfolds >90th percentile that equated to an odds ratio (OR) of 1·75 using the same methods as those used by the International Association of Diabetes and Pregnancy Study Groups (IADPSG).[4] We estimated the ORs of these outcomes at mean glucose levels and the ORs at 0·1mmol/L intervals across the full range of fasting and 2 h post-load glucose. We then plotted this range of ORs and used the plots to estimate the thresholds of fasting, and 2 h post-load glucose that were closest to ORs for each outcome

of 1·75 in both ethnic groups. These analyses were done with adjustment for the same potential confounders as in all multivariable regression analyses.

Using multivariate multiple imputation to deal with missing data
To maximise statistical power and minimise bias due to excluding women with any missing data, we used multivariate multiple imputation, with chained equations to impute missing values for covariables and outcomes for the main analyses.[5] We generated 50 imputed datasets and combined these using Rubin's rules, using the 'mi' commands in Stata.[6] Distributions of variables from pooling of the datasets with imputed variables were similar to those for observed variables (Table S1).

References

1. Office of National Statistics. Ethnic group statistics: A guide for the collection and classification of ethnicity data. file:///H:/MyDocuments/Downloads/ethnic-group-statistics_tcm77-186499pdf. 2003: Last accessed June 2015.
2. Lawlor DA, West J, Fairley L, et al. Pregnancy glycaemia and cord-blood levels of insulin and leptin in Pakistani and white British mother–offspring pairs: findings from a prospective pregnancy cohort. *Diabetologia* 2014; 57: 2492-500.
3. Williams RL. A note on robust variance estimation for cluster-correlated data. *Biometrics* 2000; 56: 645–6.
4. Metzger BE, Gabbe SG, Persson B, et al, and the International Association of Diabetes and Pregnancy Study Groups Consensus Panel. International association of diabetes and pregnancy study groups recommendations on the diagnosis and classification of hyperglycemia in pregnancy. *Diabetes Care* 2010; 33: 676–82.
5. White IR, Royston P, Wood AM. Multiple imputation using chained equations: Issues and guidance for practice. *Statis Med* 2011; 30: 377-99.
6. StataCorp. Stata Statistical Software: Release 13. College Station, Texas: StataCorp LP; 2013.

Supplementary tables and figures

Table S1: Distributions of variables with missing data comparing observed complete case data to results from pooling the datasets with imputed variables from multiple imputation

	Level/Unit	Number (%) with missing data	Complete case	Multiple imputation
Birthweight	Mean (se) standard deviation score	1	-0·37 (0·01)	-0·37 (0·01)
Sum of skinfolds	Mean (se)	3051 (32·1)	9·82 (0·03)	9·75 (0·02)
Pre-eclampsia	%	389 (4·1)	2·5	2·5
Instrumental vaginal delivery[a]	%	7 (0·1)	12·4	12·4
Maternal BMI	Mean (se)	436 (4·6)	25·8 (0·06)	25·9 (0·06)
Maternal education	% 5+ GCSE equivalent	126 (1·3)	31·5	31·5
	% Higher than A-level equivalent		25·6	25·6
Smoking	%	15 (0·2)	17·0	17·0
Alcohol	%	36 (0·4)	20·6	20·6
Parity	% primiparious	358 (3·8)	41·7	41·4
Family history of diabetes	%	297 (3·1)	25·1	25·1
Family history of hypertension	%	306 (3·2)	27·4	27·4
Previous macrosomia	%	874 (16·4)	4·5	4·8

[a]These analyses exclude women who had a Caesarean section, therefore the N=7526.

Table S2: Maternal and infant characteristics for all pregnancies and by ethnic origin. Analyses are based on complete data for each characteristic (numbers vary by characteristic and are provided in the table)

	All women		White British		South Asian		Other	
	**N**	**Mean (SD), Median (IQR) or N (%)**	**N**	**Mean (SD), Median (IQR) or N (%)**	**N**	**Mean (SD), Median (IQR) or N (%)**	**N**	**Mean (SD), Median (IQR) or N (%)**
**Primary outcomes**								
Birthweight>90th percentile[a]	9508	592 (6·2)	3887	361 (9·3)	4821	164 (3·4)	800	67 (8·4)
Sum of skinfolds >90th percentile[b]	6458	687 (10·6)	2510	270 (10·8)	3409	365 (10·7)	539	52 (9·7)
Caesarean delivery	9509	1983 (20·9)	3888	870 (22·4)	4821	907 (18·8)	800	206 (25·8)
**Secondary outcomes**								
Pre-eclampsia	9120	229 (2·5)	3724	97 (2·6)	4629	115 (2·5)	767	17 (2·2)
Preterm delivery (<37 weeks)	9509	471 (5·0)	3888	204 (5·3)	4821	227 (4·7)	8000	40 (5·0)
Shoulder dystocia[c]	7526	105 (1·4)	3018	42 (1·4)	3914	50 (1·3)	594	13 (2·2)
Instrumental vaginal delivery[c]	7519	930 (12·4)	3015	417 (13·8)	3913	417 (10·7)	591	96 (16·2)
Intensive neonatal care	9509	412 (4·3)	3888	166 (4·3)	4821	213 (4·4)	800	33 (4·1)
**Glucose levels**								
Fasting	9509	4·4 (4·2, 4·7)	3888	4·3 (4·1, 4·6)	4821	4·5 (4·2, 4·8)	800	4·4 (4·1, 4·6)
2 hour post-load	9509	5·4 (4·7, 6·1)	3888	5·3 (4·5, 6·0)	4821	5·4 (4·8, 6·2)	800	5·3 (4·6, 6·0)
**Maternal and infant characteristics**								
Maternal age at delivery (years)	9509	27·3 (5·5)	3888	26·8 (6·1)	4821	27·7 (5·0)	800	27·4 (5·7)
Age≥35 years		1092 (11·5)		487 (12·5)		503 (10·4)		102 (12·8)
Body mass index (BMI) (at booking)	9073	25·8 (5·6)	3708	26·7 (5·9)	4596	25·2 (5·3)	769	25·7 (5·5)
Obese (BMI≥30)		1808 (19·9)		899 (24·2)		768 (16·7)		141 (18·3)
Maternal education	9383		3847		4755		781	
<5 GCSEs		2024 (21·6)		788 (20·5)		1140 (24·0)		96 (12·3)
5+ GCSEs		2954 (31·5)		1336 (34·7)		1453 (30·6)		165 (21·1)
A-level		1389 (14·8)		652 (17·0)		639 (13·4)		98 (12·6)
Higher than A-level		2402 (25·6)		739 (19·2)		1352 (28·4)		311 (39·8)

Other		614 (6·5)		332 (8·6)		171 (3·6)		111 (14·2)
Smoking status	9494		3886		4809		799	
Never		6518 (68·7)		1589 (40·9)		4428 (92·1)		501 (62·7)
Before pregnancy		1359 (14·3)		973 (25·0)		227 (4·7)		159 (19·9)
In pregnancy		1617 (17·0)		1324 (34·1)		154 (3·2)		139 (17·4)
Any alcohol during pregnancy	9477	1950 (20·6)	3875	1715 (44·3)	4805	40 (0·8)	797	195 (24·5)
Primiparity	9151	3813 (41·7)	3762	1821 (48·4)	4623	1566 (33·9)	766	426 (55·6)
Family history of diabetes	9212	2313 (25·1)	3782	508 (13·4)	4660	1657 (35·6)	770	148 (19·2)
Family history of hypertension	9203	2519 (27·4)	3774	909 (24·1)	4654	1412 (30·3)	775	198 (25·6)
Previous GDM[d]	5338	56 (1·1)	1941	19 (1·0)	3057	35 (1·1)	340	2 (0·6)
Previous macrosomia (≥4kg)[d]	4464	359 (8·0)	1662	212 (12·8)	2523	124 (4·9)	279	23 (8·2)
Gestational age at OGTT (weeks)	9509	26·3 (1·9)	3888	26·2 (1·9)	4821	26·3 (1·9)	800	26·4 (1·7)
Gestational age at delivery (weeks)	9509	39·7 (1·7)	3888	39·8 (1·8)	4821	39·6 (1·7)	800	39·7 (1·7)
Male sex	9509	4884 (51·4)	3888	2006 (51·6)	4821	2464 (51·1)	800	414 (51·8)

For maternal age, maternal BMI, gestational age at OGTT, gestational age at delivery and birthweight values are mean (SD), for maternal gestational fasting and postload glucose, values are median (IQR); for all other variables (which are categorical) values are numbers (%).

[a]90th centile using the UK-WHO growth standard

[b]Internal standardisation by ethnicity and sex

[c]These analyses exclude women who had a Caesarean section, therefore the N=7526

[d]Percentages relate to multiparous women only (N=5345)

OGTT: oral glucose tolerance test; SD: standard deviation; IQR: interquartile range

Table S3: Comparison of included and excluded women, number (%) or mean (SD)

Variable	Category/statistic	N with observed data from included	Included in study (Max N=9509)	N with observed data from the potentially eligible	Potentially eligible[a] (Max N=12044)
Ethnicity	White British	9509	3888 (40·9%)	9929	4067 (41·0%)
	South Asian		4821 (50·7%)		5015 (50·5%)
	Other		800 (8·4%)		847 (8·5%)
Maternal age	Mean (SD)	9509	27.3 (5·5)	12044	27·2 (5.5)
Maternal booking BMI	Mean (SD)	9073	25.8 (5·6)	9469	25·8 (5.6)
Birthweight	Mean (SD)	9505	3253 (548)	12044	3238 (552)
Sum of skinfolds	Mean (SD)	6458	9.82 (2·02)	8238	9.82 (2·04)
Caesarean section	No	9509	7526 (79·1%)	12044	9458 (78·5%)
	Yes		1983 (20·9%)		2586 (21·5%)

[a]Potentially eligible includes 13061 women (show in flow chart, Figure 1) minus 1017 women with GDM so that in both groups (included and potentially eligible) women who delivered a live singleton child in the Bradford Royal Infirmary and who did not have existing diabetes or reach the criteria applied in the hospital during study recruitment for gestational diabetes, are compared.

Table S4: Unadjusted associations of maternal fasting and post-load glucose with primary outcomes. N = 9509 (White British N = 3888, South Asian N = 4821)

	All women		White British		South Asian	
	**OR**	**(95% CI)**	**OR**	**(95% CI)**	**OR**	**(95% CI)**
**Outcomes by fasting glucose category[a] and per 1 SD**						
**Birthweight>90th percentile**						
1 (Reference)	1·00	-	1·00	-	1·00	-
2	1·23	(0·95, 1·58)	1·31	(0·95, 1·79)	1·15	(0·66, 2·02)
3	1·48	(1·16, 1·88)	1·84	(1·36, 2·48)	1·34	(0·81, 2·21)
4	1·62	(1·19, 2·20)	2·22	(1·50, 3·29)	1·60	(0·88, 2·90)
5	2·16	(1·59, 2·94)	2·47	(1·57, 3·86)	3·26	(1·91, 5·54)
6	3·48	(2·36, 5·13)	3·92	(1·98, 7·75)	5·71	(3·15, 10·34)
7	3·37	(1·83, 6·24)	3·77	(1·50, 9·50)	5·99	(2·49, 14·44)
Per 1 SD	1·37	(1·27, 1·49)	1·44	(1·29, 1·60)	1·67	(1·45, 1·92)
**Sum of skinfolds >90th percentile**						
1 (Reference)	1·00	-	1·00	-	1·00	-
2	1·16	(0·93, 1·46)	1·11	(0·79, 1·56)	1·34	(0·96, 1·88)
3	1·57	(1·28, 1·93)	1·55	(1·12, 2·14)	1·72	(1·27, 2·32)
4	1·97	(1·53, 2·53)	2·04	(1·34, 3·10)	2·04	(1·43, 2·90)
5	2·55	(1·97, 3·30)	2·62	(1·63, 4·20)	2·67	(1·88, 3·80)
6	4·20	(3·01, 5·87)	4·20	(2·10, 8·38)	4·15	(2·67, 6·45)
7	3·82	(2·19, 6·67)	3·57	(1·34, 9·48)	4·41	(2·16, 8·98)
Per 1 SD	1·47	(1·37, 1·57)	1·46	(1·29, 1·65)	1·47	(1·34, 1·62)
**Caesarean delivery**						
1 (Reference)	1·00	-	1·00	-	1·00	-
2	1·03	(0·90, 1·19)	1·12	(0·91, 1·37)	1·04	(0·83, 1·30)
3	1·25	(1·09, 1·43)	1·34	(1·09, 1·64)	1·32	(1·08, 1·62)
4	1·41	(1·18, 1·69)	1·60	(1·20, 2·13)	1·56	(1·21, 1·99)
5	1·49	(1·22, 1·81)	1·77	(1·26, 2·47)	1·41	(1·07, 1·85)
6	1·51	(1·14, 2·01)	2·54	(1·50, 4·29)	1·27	(0·86, 1·87)
7	2·63	(1·68, 4·10)	1·88	(0·85, 4·19)	3·43	(1·95, 6·03)
Per 1 SD	1·18	(1·12, 1·24)	1·24	(1·15, 1·35)	1·18	(1·10, 1·26)
**Outcome by 2 hour post-load glucose category[a] and per 1 SD**						
**Birthweight>90th percentile**						
1 (Reference)	1·00	-	1·00	-	1·00	-
2	0·99	(0·78, 1·27)	1·13	(0·84, 1·53)	1·09	(0·64, 1·86)
3	1·22	(0·96, 1·56)	1·25	(0·92, 1·70)	1·40	(0·83, 2·34)
4	1·54	(1·13, 2·11)	1·60	(1·07, 2·41)	2·06	(1·12, 3·78)
5	1·95	(1·45, 2·64)	1·77	(1·18, 2·66)	2·98	(1·68, 5·28)
6	2·12	(1·37, 3·29)	2·72	(1·54, 4·82)	2·30	(1·01, 5·26)
7	1·50	(0·79, 2·85)	1·66	(0·69, 4·01)	2·54	(0·94, 6·89)
Per 1 SD	1·26	(1·15, 1·38)	1·25	(1·12, 1·40)	1·46	(1·23, 1·72)
**Sum of skinfolds >90th percentile**						
1 (Reference)	1·00	-	1·00	-	1·00	-
2	1·09	(0·87, 1·38)	1·31	(0·94, 1·83)	1·01	(0·72, 1·41)
3	1·49	(1·19, 1·86)	1·27	(0·89, 1·82)	1·68	(1·23, 2·29)
4	2·16	(1·66, 2·81)	2·03	(1·31, 3·16)	2·30	(1·60, 3·31)
5	2·32	(1·77, 3·03)	2·21	(1·41, 3·46)	2·56	(1·78, 3·68)
6	2·79	(1·91, 4·08)	3·21	(1·74, 5·89)	2·50	(1·48, 4·23)
7	3·06	(1·88, 4·98)	1·99	(0·76, 5·21)	3·83	(2·13, 6·88)

Per 1 SD	1·40	(1·29, 1·51)	1·34	(1·18, 1·51)	1·47	(1·32, 1·63)
**Caesarean delivery**						
1 (Reference)	1·00	-	1·00	-	1·00	-
2	1·02	(0·89, 1·17)	0·99	(0·80, 1·22)	1·14	(0·92, 1·41)
3	1·23	(1·07, 1·42)	1·32	(1·07, 1·63)	1·15	(0·92, 1·43)
4	1·35	(1·12, 1·64)	1·23	(0·91, 1·66)	1·45	(1·10, 1·92)
5	1·29	(1·06, 1·57)	1·53	(1·14, 2·06)	1·17	(0·87, 1·57)
6	1·79	(1·34, 2·39)	2·00	(1·26, 3·15)	1·66	(1·11, 2·49)
7	1·48	(1·00, 2·20)	1·26	(0·65, 2·45)	1·60	(0·94, 2·73)
Per 1 SD	1·14	(1·09, 1·20)	1·15	(1·07, 1·25)	1·12	(1·04, 1·21)

[a]Glucose categories are defined as follows: fasting plasma glucose level – category 1, <4·3 mmol/L; category 2, 4·3 – 4·4 mmol/L; category 3, 4·5 – 4·7 mmol/L; category 4, 4·8 – 4·9 mmol/L; category 5, 5·0 – 5·2 mmol/L; category 6, 5·3 – 5·6 mmol/L; category 7, 5·7 – 6·0 mmol/L. Post-load plasma glucose level – category 1, <4·7 mmol/L; category 2, 4·7 – 5·4 mmol/L; category 3, 5·5 – 6·2 mmol/L; category 4, 6·3 – 6·6 mmol/L; category 5, 6·7 – 7·2 mmol/L; category 6, 7·3 – 7·5 mmol/L; category 7, 7·6 – 7·7 mmol/L.

Table S5: Unadjusted associations of maternal fasting and post-load glucose with secondary outcomes. N = 9509 (White British N = 3888, South Asian N = 4821)

	All women		White British		South Asian	
	**OR**	**(95% CI)**	**OR**	**(95% CI)**	**OR**	**(95% CI)**
**Outcome by fasting glucose category[a] and per 1 SD**						
**Pre-eclampsia**						
1 (Reference)	1·00	-	1·00	-	1·00	-
2	1·45	(0·98, 2·14)	1·39	(0·79, 2·44)	1·46	(0·83, 2·56)
3	1·33	(0·90, 1·95)	1·22	(0·67, 2·20)	1·24	(0·72, 2·15)
4	2·00	(1·28, 3·15)	2·66	(1·37, 5·15)	1·54	(0·81, 2·92)
5	2·29	(1·41, 3·71)	3·52	(1·69, 7·36)	1·35	(0·65, 2·79)
6	2·69	(1·42, 5·09)	1·86	(0·43, 8·03)	2·14	(0·91, 5·03)
7	3·23	(1·27, 8·23)	2·03	(0·27, 15·46)	3·71	(1·26, 10·97)
Per 1 SD	1·31	(1·15, 1·48)	1·38	(1·13, 1·69)	1·19	(1·00, 1·43)
**Preterm delivery**						
1 (Reference)	1·00	-	1·00	-	1·00	-
2	0·84	(0·65, 1·08)	0·74	(0·50, 1·09)	0·96	(0·66, 1·41)
3	0·94	(0·74, 1·20)	0·91	(0·63, 1·32)	0·97	(0·68, 1·40)
4	0·78	(0·55, 1·12)	0·96	(0·56, 1·64)	0·70	(0·42, 1·18)
5	1·01	(0·70, 1·45)	1·06	(0·57, 1·97)	1·02	(0·63, 1·67)
6	0·74	(0·40, 1·38)	0·85	(0·26, 2·78)	0·62	(0·27, 1·46)
7	1·65	(0·79, 3·48)	0·59	(0·08, 4·37)	2·10	(0·87, 5·08)
Per 1 SD	0·93	(0·84, 1·03)	0·91	(0·77, 1·08)	0·93	(0·80, 1·08)
**Shoulder dystocia[b]**						
1 (Reference)	1·00	-	1·00	-	1·00	-
2	0·89	(0·51, 1·56)	0·48	(0·17, 1·30)	1·15	(0·50, 2·61)
3	0·87	(0·50, 1·53)	1·06	(0·48, 2·33)	0·90	(0·39, 2·08)
4	1·80	(0·98, 3·30)	1·59	(0·58, 4·37)	1·43	(0·56, 3·66)
5	1·33	(0·63, 2·81)	1·52	(0·44, 5·26)	1·50	(0·56, 4·02)
6	2·32	(0·96, 5·63)	3·86	(0·86, 17·34)	1·16	(0·26, 5·25)
7	2·70	(0·63, 11·57)	-	-	5·05	(1·09, 23·46)
Per 1 SD	1·26	(1·04, 1·52)	1·26	(0·90, 1·74)	1·21	(0·91, 1·59)
**Instrumental vaginal delivery[b]**						
1 (Reference)	1·00	-	1·00	-	1·00	-
2	1·09	(0·90, 1·31)	1·03	(0·79, 1·34)	0·99	(0·74, 1·33)
3	1·03	(0·85, 1·24)	0·97	(0·73, 1·28)	1·06	(0·80, 1·40)
4	1·01	(0·78, 1·30)	0·95	(0·62, 1·45)	1·05	(0·73, 1·51)
5	1·18	(0·90, 1·56)	1·47	(0·93, 2·32)	1·22	(0·84, 1·77)
6	0·98	(0·63, 1·52)	0·96	(0·37, 2·50)	1·09	(0·63, 1·86)
7	1·33	(0·64, 2·73)	1·59	(0·52, 4·80)	1·37	(0·52, 3·59)
Per 1 SD	1·02	(0·95, 1·09)	1·05	(0·94, 1·18)	1·04	(0·93, 1·15)
**Admission to neonatal unit**						
1 (Reference)	1·00	-	1·00	-	1·00	-
2	0·88	(0·67, 1·17)	0·85	(0·57, 1·28)	0·91	(0·60, 1·39)
3	0·94	(0·72, 1·22)	0·75	(0·49, 1·16)	1·09	(0·74, 1·59)
4	1·15	(0·81, 1·61)	1·12	(0·64, 1·96)	1·20	(0·75, 1·91)
5	1·14	(0·78, 1·66)	0·64	(0·27, 1·49)	1·45	(0·90, 2·34)
6	1·26	(0·73, 2·18)	2·66	(1·16, 6·07)	0·88	(0·39, 1·96)
7	0·97	(0·35, 2·69)	-	-	1·19	(0·36, 3·94)
Per 1 SD	1·00	(0·90, 1·11)	0·94	(0·78, 1·14)	1·03	(0·89, 1·18)

Outcome by 2 hour post-load glucose category[a] and per 1 SD						
**Pre-eclampsia**						
1 (Reference)	1·00	-	1·00	-	1·00	-
2	1·16	(0·78, 1·74)	1·79	(0·95, 3·39)	0·80	(0·47, 1·37)
3	1·58	(1·07, 2·33)	2·28	(1·22, 4·29)	0·97	(0·57, 1·64)
4	1·65	(1·00, 2·71)	2·85	(1·32, 6·13)	0·99	(0·49, 1·99)
5	1·86	(1·13, 3·05)	3·03	(1·40, 6·53)	0·95	(0·46, 1·99)
6	1·51	(0·67, 3·40)	2·37	(0·67, 8·32)	1·05	(0·36, 3·07)
7	2·78	(1·22, 6·31)	1·65	(0·21, 12·77)	2·30	(0·86, 6·15)
Per 1 SD	1·26	(1·11, 1·43)	1·36	(1·14, 1·64)	1·10	(0·91, 1·34)
**Preterm delivery**						
1 (Reference)	1·00	-	1·00	-	1·00	-
2	0·99	(0·77, 1·28)	1·00	(0·69, 1·44)	1·06	(0·72, 1·56)
3	0·95	(0·72, 1·24)	0·93	(0·62, 1·38)	0·97	(0·65, 1·45)
4	1·14	(0·80, 1·62)	1·14	(0·67, 1·93)	1·26	(0·76, 2·09)
5	0·99	(0·68, 1·44)	0·81	(0·44, 1·50)	1·08	(0·63, 1·84)
6	1·43	(0·86, 2·40)	1·02	(0·40, 2·60)	1·81	(0·93, 3·51)
7	0·83	(0·36, 1·93)	1·99	(0·76, 5·19)	0·25	(0·03, 1·82)
Per 1 SD	1·04	(0·94, 1·14)	1·02	(0·89, 1·17)	1·05	(0·91,1·20)
**Shoulder dystocia[b]**						
1 (Reference)	1·00	-	1·00	-	1·00	-
2	1·00	(0·55, 1·83)	1·14	(0·50, 2·60)	1·19	(0·39, 3·65)
3	1·68	(0·96, 2·95)	1·20	(0·51, 2·85)	3·19	(1·19, 8·59)
4	1·86	(0·90, 3·82)	1·71	(0·59, 4·97)	2·49	(0·72, 8·66)
5	1·74	(0·83, 3·65)	0·76	(0·17, 3·46)	3·94	(1·28, 12·13)
6	1·58	(0·47, 5·37)	1·33	(0·17, 10·51)	3·01	(0·58, 15·68)
7	3·67	(1·23, 10·93)	2·20	(0·28, 17·49)	8·09	(1·89, 34·62)
Per 1 SD	1·36	(1·12, 1·66)	1·16	(0·86, 1·55)	1·72	(1·27, 2·31)
**Instrumental vaginal delivery[b]**						
1 (Reference)	1·00	-	1·00	-	1·00	-
2	0·86	(0·71, 1·04)	0·95	(0·72, 1·25)	0·84	(0·63, 1·12)
3	0·98	(0·81, 1·19)	1·06	(0·80, 1·42)	0·96	(0·72, 1·29)
4	1·15	(0·89, 1·50)	1·18	(0·80, 1·76)	1·25	(0·86, 1·83)
5	1·05	(0·81, 1·38)	1·26	(0·84, 1·90)	0·99	(0·66, 1·47)
6	1·35	(0·90, 2·05)	2·09	(1·13, 3·85)	1·23	(0·68, 2·20)
7	1·12	(0·64, 1·97)	0·77	(0·27, 2·20)	1·46	(0·72, 2·96)
Per 1 SD	1·07	(0·99, 1·14)	1·11	(1·00, 1·23)	1·06	(0·95, 1·18)
**Admission to neonatal unit**						
1 (Reference)	1·00	-	1·00	-	1·00	-
2	0·89	(0·68, 1·16)	1·12	(0·75, 1·66)	0·70	(0·47, 1·04)
3	0·85	(0·64, 1·13)	0·81	(0·52, 1·27)	0·79	(0·53, 1·16)
4	0·94	(0·64, 1·38)	0·85	(0·45, 1·62)	0·87	(0·52, 1·46)
5	0·84	(0·56, 1·25)	0·83	(0·42, 1·61)	0·85	(0·50, 1·44)
6	0·55	(0·25, 1·20)	0·97	(0·34, 2·76)	0·35	(0·11, 1·14)
7	1·48	(0·76, 2·90)	1·38	(0·42, 4·60)	1·31	(0·54, 3·13)
Per 1 SD	0·98	(0·88, 1·08)	0·97	(0·83, 1·12)	0·98	(0·85, 1·13)

[a]Glucose categories are defined as follows: fasting plasma glucose level – category 1, <4·3 mmol/L; category 2, 4·3 – 4·4 mmol/L; category 3, 4·5 – 4·7 mmol/L; category 4, 4·8 – 4·9 mmol/L; category 5, 5·0 – 5·2 mmol/L; category 6, 5·3 – 5·6 mmol/L; category 7, 5·7 – 6·0 mmol/L. Post-load plasma glucose level – category 1, <4·7 mmol/L; category 2, 4·7 – 5·4 mmol/L; category 3, 5·5 – 6·2 mmol/L; category 4, 6·3 – 6·6 mmol/L; category 5, 6·7 – 7·2 mmol/L; category 6, 7·3 – 7·5 mmol/L; category 7, 7·6 – 7·7 mmol/L.

[b]These analyses exclude women who had a Caesarean section, therefore the N=7526.

Table S6: Confounder adjusted associations of maternal fasting and post-load glucose with secondary outcomes. N=9509 (White British N=3888, South Asian N=4821)

	**All women**		**White British**		**South Asian**		
	**OR**	**(95% CI)**	**OR**	**(95% CI)**	**OR**	**(95% CI)**	**P-interaction[b]**
**Outcome by fasting glucose category[a] and per 1 SD**							
**Pre-eclampsia**							
1 (Reference)	1·00	-	1·00	-	1·00	-	0·62
2	1·37	(0·92, 2·04)	1·28	(0·72, 2·27)	1·38	(0·78, 2·46)	
3	1·17	(0·78, 1·75)	1·08	(0·58, 1·99)	1·07	(0·60, 1·91)	
4	1·57	(0·97, 2·53)	1·88	(0·94, 3·75)	1·28	(0·65, 2·50)	
5	1·88	(1·11, 3·19)	2·65	(1·18, 5·93)	1·16	(0·53, 2·52)	
6	1·99	(1·02, 3·87)	1·16	(0·31, 4·39)	1·55	(0·63, 3·82)	
7	2·60	(0·97, 6·97)	1·65	(0·19, 14·58)	2·76	(0·86, 8·91)	
Per 1 SD	1·20	(1·04, 1·38)	1·24	(0·98, 1·55)	1·10	(0·90, 1·33)	
**Preterm delivery**							
1 (Reference)	1·00	-	1·00	-	1·00	-	0·72
2	0·86	(0·66, 1·12)	0·76	(0·52, 1·12)	0·97	(0·66, 1·42)	
3	1·02	(0·79, 1·31)	1·04	(0·71, 1·51)	1·02	(0·71, 1·47)	
4	0·86	(0·60, 1·25)	1·15	(0·67, 1·99)	0·74	(0·44, 1·24)	
5	1·15	(0·79, 1·67)	1·30	(0·69, 2·45)	1·09	(0·66, 1·81)	
6	0·83	(0·43, 1·58)	1·05	(0·31, 3·51)	0·68	(0·28, 1·66)	
7	2·12	(0·98, 4·57)	0·82	(0·10, 6·33)	2·30	(0·91, 5·82)	
Per 1 SD	0·96	(0·86, 1·08)	0·98	(0·82, 1·17)	0·95	(0·81, 1·10)	
**Shoulder dystocia[c]**							
1 (Reference)	1·00	-	1·00	-	1·00	-	0·52
2	0·88	(0·50, 1·54)	0·43	(0·15, 1·17)	1·18	(0·52, 2·72)	
3	0·87	(0·49, 1·53)	0·90	(0·41, 1·96)	0·92	(0·39, 2·21)	
4	1·69	(0·90, 3·16)	1·26	(0·47, 3·35)	1·40	(0·53, 3·65)	
5	1·19	(0·55, 2·58)	1·13	(0·33, 3·87)	1·38	(0·49, 3·86)	
6	2·01	(0·79, 5·12)	2·85	(0·69, 11·72)	0·98	(0·21, 4·64)	
7	2·56	(0·59, 11·10)	-	-	4·49	(0·92, 21·87)	
Per 1 SD	1·22	(1·00, 1·49)	1·13	(0·82, 1·56)	1·17	(0·88, 1·55)	
**Instrumental vaginal delivery[c]**							
1 (Reference)	1·00	-	1·00	-	1·00	-	0·87
2	1·16	(0·96, 1·42)	1·08	(0·81, 1·43)	1·06	(0·77, 1·45)	
3	1·15	(0·94, 1·40)	1·01	(0·75, 1·36)	1·18	(0·87, 1·60)	
4	1·18	(0·89, 1·57)	0·93	(0·59, 1·47)	1·30	(0·87, 1·93)	
5	1·53	(1·12, 2·08)	1·61	(0·97, 2·68)	1·54	(1·02, 2·33)	
6	1·27	(0·78, 2·06)	0·94	(0·33, 2·69)	1·43	(0·78, 2·62)	
7	2·21	(0·92, 5·29)	2·11	(0·51, 8·77)	2·44	(0·78, 7·68)	
Per 1 SD	1·11	(1·02, 1·20)	1·07	(0·94, 1·22)	1·13	(1·00, 1·27)	
**Intensive neonatal care**							
1 (Reference)	1·00	-	1·00	-	1·00	-	0·13
2	0·90	(0·68, 1·19)	0·87	(0·58, 1·30)	0·93	(0·61, 1·43)	
3	0·96	(0·73, 1·25)	0·78	(0·50, 1·20)	1·15	(0·79, 1·68)	
4	1·17	(0·82, 1·66)	1·17	(0·67, 2·03)	1·29	(0·80, 2·08)	
5	1·16	(0·79, 1·70)	0·64	(0·27, 1·50)	1·58	(0·97, 2·56)	
6	1·23	(0·70, 2·17)	2·47	(1·06, 5·75)	0·96	(0·42, 2·21)	
7	1·04	(0·37, 2·91)	-	-	1·33	(0·40, 4·45)	
Per 1 SD	1·00	(0·89, 1·12)	0·93	(0·77, 1·13)	1·05	(0·91, 1·22)	

	All women		White British		South Asian		
	OR	(95% CI)	OR	(95% CI)	OR	(95% CI)	P-interaction[b]
**Outcome by 2 hour post-load glucose category[a] and per 1 SD**							
**Pre-eclampsia**							
1 (Reference)	1·00	-	1·00	-	1·00	-	0.33
2	0·99	(0·65, 1·49)	1·56	(0·82, 2·98)	0·64	(0·37, 1·12)	
3	1·27	(0·84 1·90)	1·77	(0·92, 3·37)	0·78	(0·45, 1·36)	
4	1·21	(0·72, 2·06)	2·29	(1·03, 5·08)	0·66	(0·32, 1·36)	
5	1·34	(0·80, 2·25)	2·02	(0·92, 4·43)	0·66	(0·31, 1·43)	
6	1·03	(0·44, 2·41)	1·43	(0·38, 5·32)	0·70	(0·23, 2·12)	
7	2·13	(0·91, 4·97)	1·09	(0·14, 8·74)	1·77	(0·65, 4·83)	
Per 1 SD	1·13	(0·99, 1·30)	1·19	(0·99, 1·45)	0·98	(0·80, 1·21)	
**Preterm delivery**							
1 (Reference)	1·00	-	1·00	-	1·00	-	0.61
2	1·02	(0·78, 1·32)	1·07	(0·73, 1·56)	1·02	(0·69, 1·51)	
3	1·00	(0·76, 1·31)	1·02	(0·68, 1·54)	0·96	(0·64, 1·44)	
4	1·21	(0·84, 1·73)	1·25	(0·73, 2·14)	1·26	(0·75, 2·11)	
5	1·09	(0·75, 1·58)	0·96	(0·52, 1·78)	1·14	(0·67, 1·94)	
6	1·58	(0·93, 2·68)	1·25	(0·48, 3·25)	1·87	(0·95, 3·68)	
7	0·90	(0·39, 2·09)	2·13	(0·81, 5·60)	0·28	(0·04, 2·09)	
Per 1 SD	1·07	(0·97, 1·18)	1·08	(0·93, 1·24)	1·07	(0·93, 1·24)	
**Shoulder dystocia[c]**							
1 (Reference)	1·00	-	1·00	-	1·00	-	0.33
2	0·98	(0·53, 1·79)	1·13	(0·49, 2·57)	1·10	(0·36, 3·39)	
3	1·61	(0·90, 2·88)	1·07	(0·44, 2·61)	3·02	(1·09, 8·35)	
4	1·74	(0·82, 3·69)	1·39	(0·45, 4·27)	2·36	(0·64, 8·65)	
5	1·57	(0·74, 3·35)	0·53	(0·12, 2·34)	3·80	(1·21, 11·97)	
6	1·37	(0·39, 4·83)	0·82	(0·09, 7·55)	2·73	(0·53, 14·03)	
7	3·47	(1·15, 10·50)	1·81	(0·26, 12·67)	9·05	(2·00, 40·91)	
Per 1 SD	1·33	(1·08, 1·64)	1·05	(0·79, 1·40)	1·75	(1·27, 2·41)	
**Instrumental vaginal delivery[c]**							
1 (Reference)	1·00	-	1·00	-	1·00	-	0.53
2	0·81	(0·67, 1·00)	0·90	(0·67, 1·21)	0·75	(0·55, 1·03)	
3	0·96	(0·78, 1·18)	0·98	(0·72, 1·33)	0·94	(0·69, 1·30)	
4	1·15	(0·86, 1·53)	1·09	(0·71, 1·68)	1·35	(0·88, 2·06)	
5	1·01	(0·74, 1·36)	1·14	(0·71, 1·84)	0·97	(0·62, 1·50)	
6	1·46	(0·94, 2·25)	1·66	(0·86, 3·18)	1·49	(0·81, 2·73)	
7	1·00	(0·54, 1·85)	0·49	(0·16, 1·50)	1·54	(0·71, 3·34)	
Per 1 SD	1·07	(0·99, 1·15)	1·05	(0·94, 1·18)	1·10	(0·98, 1·24)	
**Intensive neonatal care**							
1 (Reference)	1·00	-	1·00	-	1·00	-	0.43
2	0·88	(0·67, 1·15)	1·16	(0·78, 1·72)	0·68	(0·46, 1·01)	
3	0·84	(0·63, 1·10)	0·83	(0·53, 1·30)	0·78	(0·53, 1·15)	
4	0·90	(0·62, 1·33)	0·83	(0·44, 1·59)	0·87	(0·51, 1·46)	
5	0·83	(0·55, 1·24)	0·85	(0·44, 1·66)	0·87	(0·51, 1·49)	
6	0·52	(0·24, 1·15)	1·03	(0·36, 2·93)	0·35	(0·11, 1·17)	
7	1·44	(0·73, 2·86)	1·27	(0·37, 4·40)	1·42	(0·58, 3·48)	
Per 1 SD	0·97	(0·88, 1·08)	0·97	(0·84, 1·13)	0·99	(0·86, 1·15)	

Models adjusted for gestational age at OGTT, presence or absence of family history of diabetes, family history of hypertension, previous GDM, previous macrosomia, smoking status, alcohol during pregnancy, mother’s age and mother’s BMI, mother’s education, baby sex and parity. Models for all

women additionally adjusted for ethnicity. Models for South Asian women not adjusted for alcohol during pregnancy because the vast majority reported never drinking alcohol. Preterm delivery additionally adjusted for squared maternal BMI because of evidence of a quadratic relationship of this with preterm delivery. Shoulder dystocia models not adjusted for previous GDM due to small numbers.

[a]Glucose categories are defined as follows: fasting plasma glucose level – category 1, <4·3 mmol/L; category 2, 4·3 – 4·4 mmol/L; category 3, 4·5 – 4·7 mmol/L; category 4, 4·8 – 4·9 mmol/L; category 5, 5·0 – 5·2 mmol/L; category 6, 5·3 – 5·6 mmol/L; category 7, 5·7 – 6·0 mmol/L. Post-load plasma glucose level – category 1, <4·7 mmol/L; category 2, 4·7 – 5·4 mmol/L; category 3, 5·5 – 6·2 mmol/L; category 4, 6·3 – 6·6 mmol/L; category 5, 6·7 – 7·2 mmol/L; category 6, 7·3 – 7·5 mmol/L; category 7, 7·6 – 7·7 mmol/L.

[b]Testing the null hypothesis that the associations of glucose categories with outcome do not differ between White British and South Asian women

[c]These analyses exclude women who had a Caesarean section, therefore the N=7526.

Tables S7: Confounder adjusted associations of fasting and post-load glucose (as continuous variables) with primary and secondary outcomes, including a squared term of the standardised glucose values to examine evidence of a quadratic effect indicative of a curvilinear association (N = 9509)

	**Fasting**		**2 hour Post-load**	
	**OR (95% CI) for 1 SD increase**	**OR (95% CI) for glucose squared**	**OR (95% CI) for 1 SD increase**	**OR (95% CI) for glucose squared**
**Primary outcomes**				
Birthweight>90th percentile	1·22 (1·08, 1·38)	1·04 (0·96, 1·13)	1·10 (0·98, 1·23)	1·04 (0·96, 1·13)
Sum of skinfolds >90th percentile	1·35 (1·18, 1·54)	1·04 (0·95, 1·14)	1·25 (1·11, 1·41)	1·04 (0·95, 1·15)
Caesarean delivery	1·06 (0·97, 1·16)	1·00 (0·95, 1·06)	1·02 (0·94, 1·10)	1·00 (0·95, 1·07)
**Secondary outcomes**				
Pre-eclampsia	1·24 (0·98, 1·57)	0·88 (0·75, 1·04)	1·26 (0·99, 1·60)	0·86 (0·72, 1·02)
Preterm delivery	0·98 (0·82, 1·17)	0·99 (0·90, 1·09)	1·08 (0·93, 1·24)	1·00 (0·90, 1·11)
Shoulder dystocia[a]	1·14 (0·82, 1·59)	0·92 (0·73, 1·17)	1·05 (0·76, 1·44)	0·93 (0·73, 1·18)
Instrumental delivery[a]	1·07 (0·94, 1·22)	0·99 (0·91, 1·07)	1·05 (0·94, 1·18)	0·99 (0·91, 1·08)
Intensive neonatal care	0·94 (0·77, 1·13)	0·99 (0·87, 1·12)	0·97 (0·83, 1·13)	0·98 (0·86, 1·12)

Models adjusted for gestational age at OGTT, presence or absence of family history of diabetes, family history of hypertension, previous GDM, previous macrosomia, smoking status, alcohol during pregnancy, mother's age and mother's BMI, mothers education, ethnicity, baby sex, parity. Birth weight>90th percentile, sum of skinfolds >90th percentile and preterm delivery additionally adjusted for squared BMI. Shoulder dystocia models not adjusted for previous GDM due to small numbers
[a]These analyses exclude women who had a Caesarean section, therefore the N=7526.

Table S8: Complete case unadjusted and confounder adjusted associations of maternal fasting and post-load glucose with primary outcomes

	**All women**				**White British**				**South Asian**			
	**Unadjusted**		**Adjusted**		**Unadjusted**		**Adjusted**		**Unadjusted**		**Adjusted**	
	**OR**	**(95% CI)**	**OR**	**(95% CI)**	**OR**	**(95% CI)**	**OR**	**(95% CI)**	**OR**	**(95% CI)**	**OR**	**(95% CI)**
**Outcome by fasting glucose category[a] and per 1 SD**												
**Birthweight >90th percentile**		(N=9508)		(N=7620)		(N=3887)		(N=3215)		(N=4821)		(N=3720)
1 (Reference)	1·00	-	1·00	-	1·00	-	1·00	-	1·00	-	1·00	-
2	1·23	(0·95, 1·58)	1·29	(0·96, 1·72)	1·31	(0·95, 1.79)	1·35	(0·95, 1.91)	1·15	(0·66, 2.02)	0·93	(0·48, 1·83)
3	1·48	(1·16, 1·88)	1·34	(1·01, 1·79)	1·84	(1·36, 2.48)	1·37	(0·97, 1.95)	1·34	(0·81, 2.21)	1·05	(0·57, 1·95)
4	1·62	(1·19, 2·20)	1·33	(0·90, 1·95)	2·22	(1·50, 3.29)	1·33	(0·82, 2.18)	1·60	(0·88, 2.90)	0·93	(0·44, 1·99)
5	2·16	(1·59, 2·94)	1·99	(1·36, 2·92)	2·47	(1·57, 3.86)	1·56	(0·.88, 2.75)	3·26	(1·91, 5.54)	2·24	(1·18, 4·24)
6	3·48	(2·36, 5·13)	3·24	(1·99, 5·26)	3·92	(1·98, 7.75)	2·34	(1·08, 5.05)	5·71	(3·15, 10.34)	3·34	(1·57, 7·08)
7	3·37	(1·83, 6·24)	2·11	(0·98, 4·54)	3·77	(1·50, 9.50)	1·98	(0·73, 5.38)	5·99	(2·49, 14.44)	2·30	(0·70, 7·58)
Per 1 SD	1·37	(1·27, 1·49)	1·27	(1·15, 1·41)	1·44	(1·29, 1.60)	1·17	(1·02, 1.34)	1·67	(1·45, 1.92)	1·41	(1·18, 1·68)
**Sum of skinfolds >90th percentile**		(N=6458)		(N=5294)		(N=2510)		(N=2101)		(N=3409)		(N=2724)
1 (Reference)	1·00	-	1·00	-	1·00	-	1·00	-	1·00	-	1·00	-
2	1·05	(0·83, 1·32)	0·95	(0·73, 1·24)	1·03	(0·71, 1·48)	0·96	(0·64, 1·44)	1·21	(0·86, 1·69)	1·06	(0·72, 1·56)
3	1·59	(1·29, 1·96)	1·54	(1·21, 1·97)	1·53	(1·09, 2·15)	1·50	(1·01, 2·22)	1·70	(1·26, 2·30)	1·60	(1·14, 2·26)
4	1·76	(1·34, 2·32)	1·47	(1·07, 2·02)	1·90	(1·20, 3·00)	1·84	(1·09, 3·09)	1·79	(1·24, 2·59)	1·32	(0·85, 2·04)
5	2·61	(1·99, 3·42)	2·05	(1·49, 2·82)	2·40	(1·45, 3·98)	2·23	(1·24, 4·01)	2·88	(2·01, 4·12)	1·98	(1·30, 3·03)
6	4·29	(3·01, 6·12)	3·53	(2·36, 5·28)	4·38	(2·16, 8·89)	3·65	(1·50, 8·91)	4·09	(2·59, 6·48)	3·27	(1·94, 5·48)
7	3·70	(2·03, 6·75)	2·92	(1·45, 5·85)	3·91	(1·37, 11·14)	3·06	(0·91, 10·27)	3·56	(1·64, 7·72)	2·82	(1·07, 7·40)
Per 1 SD	1·46	(1·36, 1·58)	1·37	(1·26, 1·49)	1·44	(1·27, 1·64)	1·39	(1·19, 1·62)	1·47	(1·33, 1·62)	1·35	(1·20, 1·51)
**Caesarean delivery**		(N=9509)		(N=7621)		(N=3888)		(N=3216)		(N=4821)		(N=3755)
1 (Reference)	1·00	-	1·00	-	1·00	-	1·00	-	1·00	-	1·00	-
2	1·03	(0·90, 1·19)	0·95	(0·81, 1·12)	1·12	(0·91, 1·37)	1·03	(0·82, 1·30)	1·04	(0·83, 1·30)	0·90	(0·70, 1·16)
3	1·25	(1·09, 1·43)	1·10	(0·94, 1·29)	1·34	(1·09, 1·64)	1·11	(0·88, 1·40)	1·32	(1·08, 1·62)	1·10	(0·87, 1·39)
4	1·41	(1·18, 1·69)	1·14	(0·93, 1·41)	1·60	(1·20, 2·13)	1·07	(0·76, 1·50)	1·56	(1·21, 1·99)	1·27	(0·95, 1·69)
5	1·49	(1·22, 1·81)	1·19	(0·94, 1·50)	1·77	(1·26, 2·47)	1·18	(0·79, 1·76)	1·41	(1·07, 1·85)	1·11	(0·81, 1·53)

6	1·51	(1·14, 2·01)	0·97	(0·68, 1·40)	2·54	(1·50, 4·29)	1·11	(0·58, 2·13)	1·27	(0·86, 1·87)	0·90	(0·55, 1·46)
7	2·63	(1·68, 4·10)	2·53	(1·54, 4·14)	1·88	(0·85, 4·19)	1·52	(0·65, 3·55)	3·43	(1·95, 6·03)	3·44	(1·80, 6·58)
Per 1 SD	1·18	(1·12, 1·24)	1·08	(1·01, 1·15)	1·24	(1·15, 1·35)	1·06	(0·96, 1·17)	1·18	(1·10, 1·26)	1·09	(1·00, 1·19)
**Outcome by 2 hour post-load glucose category[a] and per 1 SD**												
**Birthweight >90th percentile**		(N=9508)		(N=7620)		(N=3887)		(N=3215)		(N=4821)		(N=3720)
1 (Reference)	1·00	-	1·00	-	1·00	-	1·00	-	1·00	-	1·00	-
2	0·99	(0·78, 1·27)	0·85	(0·64, 1·14)	1·13	(0·84, 1·53)	0·79	(0·55, 1·12)	1·09	(0·64, 1·86)	1·07	(0·55, 2·08)
3	1·22	(0·96, 1·56)	0·99	(0·74, 1·33)	1·25	(0·92, 1·70)	0·83	(0·58, 1·19)	1·40	(0·83, 2·34)	1·27	(0·68, 2·40)
4	1·54	(1·13, 2·11)	1·19	(0·82, 1·74)	1·61	(1·07, 2·42)	0·98	(0·60, 1·58)	2·06	(1·12, 3·78)	1·74	(0·81, 3·70)
5	1·95	(1·45, 2·64)	1·45	(1·01, 2·09)	1·77	(1·18, 2·66)	1·07	(0·67, 1·71)	2·98	(1·68, 5·28)	2·15	(1·02, 4·54)
6	2·12	(1·37, 3·29)	1·82	(1·08, 3·07)	2·72	(1·54, 4·82)	1·78	(0·91, 3·47)	2·30	(1·01, 5·26)	2·60	(1·02, 6·62)
7	1·50	(0·79, 2·85)	1·31	(0·60, 2·85)	1·66	(0·69, 4·01)	0·85	(0·27, 2·68)	2·54	(0·94, 6·89)	2·59	(0·82, 8·19)
Per 1 SD	1·26	(1·15, 1·38)	1·15	(1·03, 1·29)	1·25	(1·12, 1·40)	1·06	(0·93, 1·22)	1·46	(1·23, 1·72)	1·35	(1·08, 1·68)
**Sum of skinfolds >90th percentile**		(N=6458)		(N=5294)		(N=2510)		(N=2101)		(N=3409)		(N=2724)
1 (Reference)	1·00	-	1·00	-	1·00	-	1·00	-	1·00	-	1·00	-
2	1·02	(0·81, 1·29)	1·05	(0·80, 1·37)	1·31	(0·91, 1·89)	1·32	(0·88, 1·98)	0·91	(0·65, 1·29)	0·94	(0·63, 1·40)
3	1·44	(1·15, 1·81)	1·35	(1·04, 1·76)	1·16	(0·79, 1·70)	0·98	(0·63, 1·52)	1·65	(1·20, 2·28)	1·63	(1·12, 2·36)
4	2·26	(1·72, 2·.97)	1·97	(1·44, 2·70)	2·09	(1·32, 3·29)	1·96	(1·17, 3·26)	2·50	(1·71, 3·64)	2·15	(1·38, 3·34)
5	2·21	(1·67, 2·92)	1·89	(1·37, 2·61)	2·36	(1·49, 3·74)	2·13	(1·27, 3·58)	2·41	(1·65, 3·53)	2·08	(1·32, 3·29)
6	2·91	(1·98, 4·29)	2·34	(1·48, 3·69)	3·54	(1·84, 6·82)	3·42	(1·59, 7·36)	2·68	(1·59, 4·50)	2·01	(1·08, 3·75)
7	3·04	(1·87, 4·94)	2·62	(1·51, 4·55)	1·96	(0·73, 5·26)	2·01	(0·68, 5·90)	3·81	(2·09, 6·94)	3·24	(1·62, 6·47)
Per 1 SD	1·41	(1·31, 1·53)	1·31	(1·20, 1·44)	1·36	(1·20, 1·55)	1·31	(1·13, 1·52)	1·49	(1·34, 1·66)	1·36	(1·20, 1·55)
**Caesarean delivery**		(N=9509)		(N=7621)		(N=3888)		(N=3216)		(N=4821)		(N=3755)
1 (Reference)	1·00	-	1·00	-	1·00	-	1·00	-	1·00	-	1·00	-
2	1·02	(0·89, 1·17)	0·94	(0·80, 1·10)	0·99	(0·80, 1·22)	0·87	(0·68, 1·10)	1·14	(0·92, 1·41)	1·09	(0·85, 1·39)
3	1·23	(1·07, 1·42)	1·04	(0·88, 1·22)	1·32	(1·07, 1·63)	1·07	(0·84, 1·36)	1·15	(0·92, 1·43)	1·05	(0·81, 1·35)
4	1·35	(1·12, 1·64)	1·15	(0·92, 1·43)	1·23	(0·91, 1·66)	0·88	(0·62, 1·25)	1·45	(1·10, 1·92)	1·36	(0·98, 1·89)
5	1·29	(1·06, 1·57)	1·03	(0·82, 1·29)	1·53	(1·14, 2·06)	1·02	(0·72, 1·43)	1·17	(0·87, 1·57)	1·07	(0·76, 1·51)

6	1·79	(1·34, 2·39)	1·30	(0·92, 1·83)	2·00	(1·26, 3·15)	0·96	(0·55, 1·69)	1·66	(1·11, 2·49)	1·57	(0·99, 2·48)
7	1·48	(1·00, 2·20)	1·04	(0·65, 1·66)	1·26	(0·65, 2·45)	0·62	(0·27, 1·42)	1·60	(0·94, 2·73)	1·36	(0·74, 2·53)
Per 1 SD	1·14	(1·09, 1·20)	1·05	(0·99, 1·12)	1·15	(1·07, 1·25)	1·00	(0·91, 1·09)	1·12	(1·04, 1·21)	1·09	(1·00, 1·19)

[a]Glucose categories are defined as follows: fasting plasma glucose level – category 1, <4·3 mmol/L; category 2, 4·3 – 4·4 mmol/L; category 3, 4·5 – 4·7 mmol/L; category 4, 4·8 – 4·9 mmol/L; category 5, 5·0 – 5·2 mmol/L; category 6, 5·3 – 5·6 mmol/L; category 7, 5·7 – 6·0 mmol/L. Post-load plasma glucose level – category 1, <4·7 mmol/L; category 2, 4·7 – 5·4 mmol/L; category 3, 5·5 – 6·2 mmol/L; category 4, 6·3 – 6·6 mmol/L; category 5, 6·7 – 7·2 mmol/L; category 6, 7·3 – 7·5 mmol/L; category 7, 7·6 – 7·7 mmol/L.

Tables S9: Complete case unadjusted and confounder adjusted associations of maternal fasting and post-load glucose with secondary outcomes

	All women				White British				South Asian			
	Unadjusted		Adjusted		Unadjusted		Adjusted		Unadjusted		Adjusted	
	**OR**	**(95% CI)**	**OR**	**(95% CI)**	**OR**	**(95% CI)**	**OR**	**(95% CI)**	**OR**	**(95% CI)**	**OR**	**(95% CI)**
**Outcome by fasting glucose category[a] and per 1 SD**												
**Pre-eclampsia**		(N=9120)		(N=7407)		(N=3724)		(N=3135)		(N=4629)		(N=3610)
1 (Reference)	1·00	-	1·00	-	1·00	-	1·00	-	1·00	-	1·00	-
2	1·49	(1·01, 2·20)	1·32	(0·87, 2·00)	1·43	(0·81, 2·52)	1·36	(0·75, 2·48)	1·48	(0·84, 2·60)	1·19	(0·65, 2·16)
3	1·35	(0·91, 1·99)	1·14	(0·74, 1·77)	1·23	(0·68, 2·24)	1·16	(0·61, 2·21)	1·26	(0·73, 2·18)	0·88	(0·48, 1·63)
4	1·.99	(1·27, 3·12)	1·51	(0·90, 2·51)	2·70	(1·40, 5·23)	1·93	(0·94, 3·97)	1·47	(0·78, 2·80)	1·07	(0·51, 2·24)
5	2·37	(1·46, 3·84)	1·85	(1·06, 3·23)	3·64	(1·74, 7·63)	2·54	(1·09, 5·94)	1·37	(0·66, 2·83)	1·01	(0·44, 2·34)
6	2·68	(1·41, 5·08)	1·47	(0·67, 3·23)	1·88	(0·44, 8·14)	1·28	(0·34, 4·86)	2·07	(0·88, 4·89)	0·96	(0·32, 2·91)
7	3·30	(1·29, 8·39)	3·02	(1·09, 8·32)	1·99	(0·26, 15·20)	1·47	(0·16, 13·70)	3·81	(1·29, 11·25)	3·74	(1·10, 12·68)
Per 1 SD	1·31	(1·16, 1·48)	1·19	(1·02, 1·38)	1·38	(1·13, 1·69)	1·22	(0·97, 1·53)	1·19	(0·99, 1·42)	1·07	(0·85, 1·34)
**Preterm delivery**		(N=9509)		(N=7621)		(N=3888)		(N=3198)		(N=4821)		(N=3755)
1 (Reference)	1·00	-	1·00	-	1·00	-	1·00	-	1·00	-	1·00	-
2	0·84	(0·65, 1·08)	0·82	(0·62, 1·09)	0·74	(0·50, 1·09)	0·77	(0·51, 1·18)	0·96	(0·66, 1·41)	0·91	(0·60, 1·39)
3	0·94	(0·74, 1·20)	1·04	(0·79, 1·37)	0·91	(0·63, 1·32)	1·04	(0·69, 1·57)	0·97	(0·68, 1·40)	1·04	(0·70, 1·55)
4	0·78	(0·55, 1·12)	0·89	(0·59, 1·33)	0·96	(0·56, 1·64)	1·19	(0·65, 2·21)	0·70	(0·42, 1·18)	0·76	(0·43, 1·34)
5	1·01	(0·70, 1·45)	1·29	(0·86, 1·94)	1·06	(0·57, 1·97)	1·40	(0·69, 2·83)	1·02	(0·63, 1·67)	1·20	(0·70, 2·06)
6	0·74	(0·40, 1·38)	0·75	(0·36, 1·58)	0·85	(0·26, 2·78)	0·86	(0·20, 3·69)	0·62	(0·27, 1·46)	0·56	(0·20, 1·59)
7	1·65	(0·79, 3·48)	2·30	(1·01, 5·25)	0·59	(0·08, 4·37)	1·09	(0·14, 8·72)	2·10	(0·87, 5·08)	2·52	(0·92, 6·93)
Per 1 SD	0·93	(0·84, 1·03)	0·99	(0·87, 1·11)	0·91	(0·77, 1·08)	1·00	(0·82, 1·22)	0·93	(0·80, 1·08)	0·94	(0·80, 1·11)
**Shoulder dystocia**		(N=7526)		(N=6040)		(N=2998)		(N=2496)		(N=3914)		(N=3015)
1 (Reference)	1·00	-	1·00	-	1·00	-	1·00	-	1·00	-	1·00	-
2	0·89	(0·51, 1·56)	0·98	(0·52, 1·86)	0·48	(0·17, 1·30)	0·52	(0·16, 1·65)	1·15	(0·50, 2·61)	1·09	(0·44, 2·70)
3	0·87	(0·50, 1·53)	1·05	(0·55, 1·99)	1·06	(0·48, 2·33)	1·36	(0·59, 3·17)	0·90	(0·39, 2·08)	0·77	(0·29, 2·08)
4	1·80	(0·98, 3·30)	2·17	(1·09, 4·32)	1·59	(0·58, 4·37)	1·90	(0·67, 5·44)	1·43	(0·56, 3·66)	1·46	(0·54, 3·97)
5	1·33	(0·63, 2·81)	1·00	(0·37, 2·73)	1·52	(0·44, 5·26)	1·19	(0·24, 5·75)	1·50	(0·56, 4·02)	0·90	(0·24, 3·34)
6	2·32	(0·96, 5·63)	3·00	(1·15, 7·84)	3·86	(0·86, 17·34)	3·59	(0·81, 15·83)	1·16	(0·26, 5·25)	1·34	(0·28, 6·53)
7	2·70	(0·63, 11·57)	2·08	(0·27, 16·12)	-	-	-	-	5·05	(1·09, 23·46)	3·57	(0·43, 30·02)
Per 1 SD	1·26	(1·04, 1·52)	1·28	(1·03, 1·60)	1·26	(0·90, 1·74)	1·27	(0·91, 1·77)	1·21	(0·91, 1·59)	1·12	(0·81, 1·56)

**Instrumental vaginal delivery**		(N=7519)		(N=6034)		(N=3015)		(N=2509)		(N=3913)		(N=3043)
1 (Reference)	1·00	-	1·00	-	1·00	-	1·00	-	1·00	-	1·00	-
2	1·09	(0·90, 1·31)	1·22	(0·99, 1·50)	1·03	(0·78, 1·34)	1·10	(0·82, 1·48)	0·99	(0·74, 1·33)	1·08	(0·76, 1·52)
3	1·02	(0·85, 1·24)	1·17	(0·94, 1·46)	0·97	(0·73, 1·28)	1·04	(0·76, 1·43)	1·05	(0·79, 1·40)	1·23	(0·88,1·71)
4	1·01	(0·78, 1·30)	1·30	(0·96, 1·75)	0·95	(0·62, 1·45)	1·05	(0·65, 1·69)	1·05	(0·73, 1·51)	1·41	(0·92, 2·16)
5	1·18	(0·90, 1·55)	1·63	(1·17, 2·28)	1·46	(0·92, 2·31)	1·83	(1·08, 3·12)	1·22	(0·84, 1·77)	1·58	(0·99, 2·51)
6	0·98	(0·63, 1·52)	1·21	(0·72, 2·06)	0·96	(0·37, 2·50)	1·02	(0·35, 2·94)	1·09	(0·63, 1·86)	1·30	(0·67, 2·53)
7	1·33	(0·64, 2·73)	1·77	(0·64, 4·92)	1·59	(0·52, 4·80)	0·98	(0·17, 5·58)	1·37	(0·52, 3·59)	3·14	(0·89,11·12)
Per 1 SD	1·02	(0·95, 1·09)	1·11	(1·01, 1·21)	1·05	(0·94, 1·18)	1·08	(0·95, 1·24)	1·04	(0·93, 1·15)	1·12	(0·98,1·28)
**Admission to neonatal unit**		(N=9509)		(N=7621)		(N=3859)		(N=3174)		(N=4821)		(N=3755)
1 (Reference)	1·00	-	1·00	-	1·00	-	1·00	-	1·00	-	1·00	-
2	0·88	(0·67, 1·17)	0·90	(0·66, 1·21)	0·85	(0·57, 1·28)	0·89	(0·58, 1·37)	0·91	(0·60, 1·39)	0·95	(0·60, 1·49)
3	0·94	(0·72, 1·22)	0·89	(0·66, 1·20)	0·75	(0·49, 1·16)	0·77	(0·48, 1·23)	1·09	(0·74, 1·59)	1·09	(0·72, 1·65)
4	1·15	(0·81, 1·61)	0·97	(0·65, 1·46)	1·12	(0·64, 1·96)	1·12	(0·60, 2·12)	1·20	(0·75, 1·91)	1·03	(0·60, 1·78)
5	1·14	(0·78, 1·66)	1·29	(0·86, 1·94)	0·64	(0·27, 1·49)	0·68	(0·26, 1·72)	1·45	(0·90, 2·34)	1·77	(1·07, 2·93)
6	1·26	(0·73, 2·18)	1·09	(0·57, 2·10)	2·66	(1·16, 6·07)	1·90	(0·65, 5·60)	0·88	(0·39, 1·96)	0·94	(0·39, 2·25)
7	0·97	(0·35, 2·69)	0·91	(0·28, 2·93)	-	-	-	-	1·19	(0·36, 3·94)	1·05	(0·25, 4·49)
Per 1 SD	1·00	(0·90, 1·11)	0·97	(0·86, 1·10)	0·94	(0·78, 1·14)	0·91	(0·74, 1·12)	1·03	(0·89, 1·18)	1·02	(0·87, 1·20)
**Outcome by 2 hour post-load glucose category[a] and per 1 SD**												
**Pre-eclampsia**		(N=9120)		(N=7407)		(N=3724)		(N=3135)		(N=4629)		(N=3610)
1 (Reference)	1·00	-	1·00	-	1·00	-	1·00	-	1·00	-	1·00	-
2	1·15	(0·77, 1·73)	0·92	(0·59, 1·43)	1·83	(0·97, 3·49)	1·62	(0·81, 3·22)	0·77	(0·45, 1·32)	0·54	(0·30, 0·96)
3	1·60	(1·09, 2·37)	1·25	(0·81, 1·93)	2·34	(1·24, 4·41)	2·02	(1·02, 4·01)	0·96	(0·57, 1·63)	0·67	(0·37, 1·21)
4	1·67	(1·01, 2·75)	1·36	(0·78, 2·37)	2·96	(1·37, 6·40)	3·03	(1·35, 6·81)	0·97	(0·48, 1·94)	0·61	(0·28, 1·32)
5	1·84	(1·12, 3·02)	1·38	(0·80, 2·38)	3·06	(1·42, 6·61)	2·31	(1·01, 5·28)	0·89	(0·43, 1·87)	0·56	(0·24, 1·29)
6	1·52	(0·68, 3·42)	1·10	(0·44, 2·76)	2·40	(0·68, 8·44)	1·87	(0·50, 7·08)	1·04	(0·36, 3·02)	0·63	(0·17, 2·27)
7	2·85	(1·26, 6·47)	1·67	(0·61, 4·52)	1·58	(0·20, 12·23)	1·21	(0·15, 10·01)	2·35	(0·88, 6·29)	1·06	(0·31, 3·58)
Per 1 SD	1·27	(1·11, 1·44)	1·14	(0·98, 1·33)	1·37	(1·14, 1·64)	1·27	(1·04, 1·55)	1·10	(0·90, 1·34)	0·92	(0·73, 1·16)
**Preterm delivery**		(N=9509)		(N=7621)		(N=3888)		(N=3198)		(N=4821)		(N=3755)
1 (Reference)	1·00	-	1·00	-	1·00	-	1·00	-	1·00	-	1·00	-

2	0·99	(0·77, 1·28)	0·96	(0·72, 1·27)	1·00	(0·69, 1·44)	1·03	(0·68, 1·54)	1·06	(0·72, 1·56)	0·93	(0·61, 1·43)
3	0·95	(0·72, 1·24)	0·94	(0·70, 1·27)	0·93	(0·62, 1·38)	0·94	(0·60, 1·47)	0·97	(0·65, 1·45)	0·91	(0·58, 1·42)
4	1·14	(0·80, 1·62)	1·23	(0·84, 1·81)	1·14	(0·67, 1·93)	1·12	(0·62, 2·04)	1·26	(0·76, 2·09)	1·33	(0·77, 2·29)
5	0·99	(0·68, 1·44)	1·12	(0·75, 1·67)	0·81	(0·44, 1·50)	0·93	(0·48, 1·81)	1·08	(0·63, 1·84)	1·21	(0·68, 2·15)
6	1·43	(0·86, 2·40)	1·66	(0·95, 2·90)	1·02	(0·40, 2·60)	1·14	(0·39, 3·29)	1·81	(0·93, 3·51)	2·02	(0·99, 4·10)
7	0·83	(0·36, 1·93)	1·08	(0·46, 2·56)	1·99	(0·76, 5·19)	2·64	(0·97, 7·18)	0·25	(0·03, 1·82)	0·31	(0·04, 2·39)
Per 1 SD	1·04	(0·94, 1·14)	1·09	(0·98, 1·21)	1·02	(0·89, 1·17)	1·06	(0·91, 1·24)	1·05	(0·91, 1·20)	1·11	(0·95, 1·31)
**Shoulder dystocia**		(N=7526)		(N=6040)		(N=3018)		(N=2512)		(N=3914)		(N=3015)
1 (Reference)	1·00	-	1·00	-	1·00	-	1·00	-	1·00	-	1·00	-
2	1·00	(0·55, 1·83)	1·06	(0·54, 2·10)	1·14	(0·50, 2·60)	1·10	(0·45, 2·70)	1·19	(0·39, 3·65)	1·06	(0·29, 3·81)
3	1·68	(0·96, 2·95)	1·49	(0·76, 2·90)	1·20	(0·51, 2·85)	0·84	(0·30, 2·33)	3·19	(1·19, 8·59)	2·63	(0·85, 8·09)
4	1·86	(0·90, 3·82)	1·88	(0·81, 4·37)	1·71	(0·59, 4·97)	1·24	(0·37, 4·17)	2·49	(0·72, 8·66)	3·09	(0·79, 12·05)
5	1·74	(0·83, 3·65)	1·62	(0·69, 3·81)	0·76	(0·17, 3·46)	0·56	(0·12, 2·57)	3·94	(1·28, 12·13)	3·59	(0·98, 13·09)
6	1·58	(0·47, 5·37)	1·76	(0·48, 6·45)	1·33	(0·17, 10·51)	0·77	(0·08, 7·47)	3·01	(0·58, 15·68)	3·93	(0·71, 21·77)
7	3·67	(1·23, 10·93)	3·25	(0·91, 11·57)	2.20	(0·28, 17·49)	1·77	(0·24, 12·96)	8.09	(1·89, 34·62)	6·64	(1·14, 38·68)
Per 1 SD	1·36	(1·12, 1·66)	1·32	(1·04, 1·68)	1.16	(0·86, 1·55)	1·02	(0·75, 1·39)	1.72	(1·27, 2·31)	1·73	(1·19, 2·51)
**Instrumental vaginal delivery**		(N=7519)		(N=6034)		(N=3015)		(N=2509)		(N=3913)		(N=3043)
1 (Reference)	1·00	-	1·00	-	1·00	-	1·00	-	1·00	-	1·00	-
2	0·86	(0·71, 1·04)	0·81	(0·66, 1·01)	0·95	(0·72, 1·25)	0·99	(0·73, 1·35)	0·84	(0·63, 1·12)	0·67	(0·48, 0·95)
3	0·98	(0·81, 1·19)	0·93	(0·75, 1·16)	1·06	(0·80, 1·42)	0·99	(0·71, 1·38)	0·96	(0·72, 1·29)	0·90	(0·64, 1·27)
4	1·15	(0·89, 1·49)	1·05	(0·77, 1·43)	1·18	(0·79, 1·76)	1·02	(0·64, 1·60)	1·25	(0·86, 1·83)	1·23	(0·78, 1·94)
5	1·05	(0·81, 1·38)	0·84	(0·61, 1·17)	1·26	(0·84, 1·90)	1·12	(0·68, 1·83)	0·99	(0·66, 1·47)	0·66	(0·40, 1·09)
6	1·35	(0·90, 2·05)	1·46	(0·92, 2·33)	2·09	(1·13, 3·85)	1·54	(0·77, 3·08)	1·23	(0·68, 2·20)	1·58	(0·83, 3·01)
7	1·12	(0·64, 1·97)	0·87	(0·46, 1·66)	0·77	(0·27, 2·20)	0·51	(0·16, 1·60)	1·46	(0·72, 2·96)	1·36	(0·61, 3·01)
Per 1 SD	1·06	(0·99, 1·14)	1·02	(0·94, 1·10)	1·11	(1·00, 1·23)	1·03	(0·92, 1·16)	1·06	(0·95, 1·18)	1·03	(0·91, 1·18)
**Admission to neonatal unit**		(N=9509)		(N=7621)		(N=3888)		(N=3198)		(N=4821)		(N=3755)
1 (Reference)	1·00	-	1·00	-	1·00	-	1·00	-	1·00	-	1·00	-
2	0·89	(0·68, 1·16)	0·80	(0·60, 1·08)	1·12	(0·75, 1·66)	1·19	(0·77, 1·82)	0·70	(0·47, 1·04)	0·54	(0·35, 0·84)
3	0·85	(0·64, 1·13)	0·78	(0·58, 1·06)	0·81	(0·52, 1·27)	0·86	(0·53, 1·39)	0·79	(0·53, 1·16)	0·72	(0·47, 1·09)
4	0·94	(0·64, 1·38)	0·92	(0·61, 1·38)	0·85	(0·45, 1·62)	0·92	(0·47, 1·81)	0·87	(0·52, 1·46)	0·88	(0·51, 1·52)
5	0·84	(0·56, 1·25)	0·82	(0·53, 1·26)	0·83	(0·42, 1·61)	0·73	(0·34, 1·57)	0·85	(0·50, 1·44)	0·90	(0·52, 1·56)
6	0·55	(0·25, 1·20)	0·43	(0·17, 1·08)	0·97	(0·34, 2·76)	0·91	(0·28, 3·01)	0·35	(0·11, 1·14)	0·25	(0·06, 1·06)
7	1·48	(0·76, 2·90)	1·15	(0·51, 2·57)	1·38	(0·42, 4·60)	1·10	(0·25, 4·91)	1·31	(0·54, 3·13)	1·20	(0·45, 3·19)

Per 1 SD	0·98	(0·88, 1·08)	0·95	(0·85, 1·06)	0·97	(0·83, 1·12)	0·95	(0·81, 1·11)	0·98	(0·85, 1·13)	0·99	(0·84, 1·17)

[a]Glucose categories are defined as follows: fasting plasma glucose level – category 1, <4·3 mmol/L; category 2, 4·3 – 4·4 mmol/L; category 3, 4·5 – 4·7 mmol/L; category 4, 4·8 – 4·9 mmol/L; category 5, 5·0 – 5·2 mmol/L; category 6, 5·3 – 5·6 mmol/L; category 7, 5·7 – 6·0 mmol/L. Post-load plasma glucose level – category 1, <4·7 mmol/L; category 2, 4·7 – 5·4 mmol/L; category 3, 5·5 – 6·2 mmol/L; category 4, 6·3 – 6·6 mmol/L; category 5, 6·7 – 7·2 mmol/L; category 6, 7·3 – 7·5 mmol/L; category 7, 7·6 – 7·7 mmol/L.

Table S10: Unadjusted and confounder adjusted odds ratios (95% CI) for associations between maternal fasting and post-load glucose and primary outcomes for Pakistani women only (N=4201)

	Unadjusted		Confounder adjusted	
	**OR**	**(95% CI)**	**OR**	**(95% CI)**
**Outcome by fasting glucose category[a] and per 1 SD**				
**Birth weight>90th centile**				
1 (Reference)	1·00	-	1·00	-
2	1·35	(0·75, 2·41)	1·31	(0·71, 2·41)
3	1·45	(0·85, 2·46)	1·22	(0·69, 2·14)
4	1·86	(1·01, 3·45)	1·26	(0·65, 2·43)
5	3·71	(2·12, 6·49)	2·45	(1·34, 4·46)
6	6·59	(3·53, 12·31)	3·82	(1·89, 7·70)
7	6·68	(2·72, 16·42)	3·77	(1·47, 9·66)
Per 1 SD	1·70	(1·48, 1·96)	1·45	(1·24,1·70)
**Sum of skinfolds >90th centile**				
1 (Reference)	1·00	-	1·00	-
2	1·36	(0·96, 1.93)	1·31	(0·92, 1·88)
3	1·78	(1·30, 2.45)	1·60	(1·15, 2·22)
4	2·09	(1·43, 3.04)	1·76	(1·19, 2·59)
5	2·60	(1·78, 3.80)	2·05	(1·38, 3·05)
6	4·07	(2·54, 6.53)	3·02	(1·85, 4·92)
7	4·21	(1·97, 8.99)	2·90	(1·29, 6·51)
Per 1 SD	1·45	(1·31, 1.60)	1·33	(1·19, 1·48)
**Caesarean delivery**				
1 (Reference)	1·00	-	1·00	-
2	1·05	(0·83, 1·34)	0·99	(0·78, 1·27)
3	1·30	(1·04, 1·63)	1·17	(0·93, 1·47)
4	1·59	(1·22, 2·07)	1·37	(1·04, 1·81)
5	1·46	(1·09, 1·95)	1·22	(0·90, 1·65)
6	1·28	(0·83, 1·95)	1·01	(0·64, 1·60)
7	3·23	(1·77, 5·90)	2·64	(1·39, 5·04)
Per 1 SD	1·18	(1·10, 1·28)	1·11	(1·02, 1·21)
**Outcome by 2 hour post-load glucose category[a] and per 1 SD**				
**Birth weight>90th centile**				
1 (Reference)	1·00	-	1·00	-
2	1·25	(0·71, 2·20)	1·11	(0·62, 1·96)
3	1·59	(0·92, 2·74)	1·21	(0·70, 2·12)
4	2·44	(1·30, 4·58)	1·64	(0·83, 3·22)
5	3·22	(1·76, 5·91)	2·25	(1·18, 4·30)
6	2·40	(0·99, 5·83)	1·77	(0·69, 4·54)
7	2·93	(1·06, 8·08)	1·97	(0·63, 6·11)
Per 1 SD	1·48	(1·25, 1·75)	1·30	(1·08, 1·58)
**Sum of skinfolds >90th centile**				
1 (Reference)	1·00	-	1·00	-
2	0·99	(0·69, 1·41)	0·94	(0·65, 1·34)
3	1·60	(1·16, 2·23)	1·45	(1·04, 2·01)
4	2·34	(1·60, 3·43)	1·96	(1·32, 2·90)
5	2·58	(1·77, 3·76)	2·21	(1·50, 3·27)

6	2·40	(1·37, 4·22)	2·03	(1·14, 3·61)
7	3·24	(1·70, 6·16)	2·64	(1·35, 5·14)
Per 1 SD	1·45	(1·30, 1·62)	1·36	(1·21, 1·53)
**Caesarean delivery**				
1 (Reference)	1·00	-	1·00	-
2	1·22	(0·96, 1·54)	1·16	(0·91, 1·48)
3	1·19	(0·94, 1·51)	1·07	(0·84, 1·37)
4	1·55	(1·15, 2·09)	1·31	(0·95, 1·79)
5	1·26	(0·92, 1·72)	1·04	(0·75, 1·45)
6	1·66	(1·07, 2·59)	1·41	(0·88, 2·26)
7	1·50	(0·85, 2·65)	1·15	(0·63, 2·10)
Per 1 SD fasting glucose	1·13	(1·04, 1·23)	1·06	(0·97, 1·15)

Models adjusted for gestational age at OGTT, presence or absence of family history of diabetes, previous GDM, previous macrosomia, smoking status, mother's age and mother's BMI, mothers education, baby sex and parity. Birth weight>90th percentile, sum of skinfolds >90th percentile and preterm delivery additionally adjusted for squared BMI. Shoulder dystocia models not adjusted for previous GDM due to small numbers.

[a]Glucose categories are defined as follows: fasting plasma glucose level – category 1, <4·3 mmol/L; category 2, 4·3 – 4·4 mmol/L; category 3, 4·5 – 4·7 mmol/L; category 4, 4·8 – 4·9 mmol/L; category 5, 5·0 – 5·2 mmol/L; category 6, 5·3 – 5·6 mmol/L; category 7, 5·7 – 6·0 mmol/L. Post-load plasma glucose level – category 1, <4·7 mmol/L; category 2, 4·7 – 5·4 mmol/L; category 3, 5·5 – 6·2 mmol/L; category 4, 6·3 – 6·6 mmol/L; category 5, 6·7 – 7·2 mmol/L; category 6, 7·3 – 7·5 mmol/L; category 7, 7·6 – 7·7 mmol/L.

Table S11: Unadjusted and confounder adjusted odds ratios (95% CI) for associations between maternal fasting and post-load glucose and secondary outcomes for Pakistani women only (N=4201)

	**Unadjusted**		**Confounder adjusted**	
	**OR**	**(95% CI)**	**OR**	**(95% CI)**
**Outcome by fasting glucose category[a] and per 1 SD**				
**Pre-eclampsia**				
1 (Reference)	1·00	-	1·00	-
2	1·41	(0·78, 2·55)	1·31	(0·72, 2·41)
3	1·24	(0·70, 2·19)	1·06	(0·58, 1·92)
4	1·57	(0·81, 3·04)	1·25	(0·62, 2·52)
5	1·48	(0·71, 3·11)	1·18	(0·53, 2·63)
6	1·40	(0·48, 4·08)	0·99	(0·34, 2·89)
7	3·86	(1·30, 11·51)	2·70	(0·78, 9·38)
Per 1 SD	1·16	(0·96, 1·41)	1·06	(0·86, 1·29)
**Preterm delivery**				
1 (Reference)	1·00	-	1·00	-
2	1·01	(0·66, 1·53)	1·01	(0·66, 1·54)
3	1·04	(0·70, 1·54)	1·09	(0·73, 1·62)
4	0·70	(0·39, 1·24)	0·72	(0·40, 1·29)
5	1·18	(0·70, 1·99)	1·25	(0·73, 2·14)
6	0·67	(0·26, 1·70)	0·72	(0·27, 1·93)
7	2·52	(1·03, 6·18)	2·74	(1·08, 6·97)
Per 1 SD fasting glucose	0·98	(0·84, 1·15)	1·00	(0·85, 1·18)
**Shoulder dystocia[b]**				
1 (Reference)	1·00	-	1·00	-
2	1·13	(0·48, 2·67)	1·16	(0·49, 2·78)
3	0·66	(0·26, 1·72)	0·69	(0·26, 1·85)
4	1·33	(0·49, 3·60)	1·31	(0·47, 3·61)
5	1·08	(0·34, 3·43)	1·04	(0·33, 3·27)
6	1·29	(0·28, 5·89)	1·06	(0·22, 5·21)
7	5·05	(1·07, 23·74)	4·44	(0·86, 22·82)
Per 1 SD fasting glucose	1·17	(0·85, 1·62)	1·14	(0·82, 1·58)
**Instrumental vaginal delivery[b]**				
1 (Reference)	1·00	-	1·00	-
2	0·95	(0·68, 1·31)	0·99	(0.70, 1.40)
3	1·03	(0·75, 1·40)	1·11	(0.79, 1.55)
4	1·15	(0·78, 1·69)	1·33	(0.87, 2.03)
5	1·03	(0·67, 1·58)	1·25	(0.78, 2.01)
6	1·29	(0·73, 2·28)	1·77	(0.93, 3.37)
7	1·55	(0·58, 4·11)	2·45	(0.78, 7.74)
Per 1 SD fasting glucose	1·05	(0·94, 1·18)	1·14	(1.00, 1.30)
**Intensive neonatal care**				
1 (Reference)	1.00	-	1·00	-
2	0.87	(0.55, 1.38)	0·87	(0·55, 1·39)
3	1.09	(0.73, 1.64)	1·13	(0·76, 1·69)
4	1.24	(0.75, 2.05)	1·29	(0·77, 2·15)
5	1.45	(0.86, 2.43)	1·52	(0·90, 2·57)
6	1.06	(0.47, 2.39)	1·11	(0·47, 2·59)
7	1.33	(0.40, 4.42)	1·38	(0·41, 4·63)
Per 1 SD fasting glucose	1.06	(0.91, 1.24)	1·07	(0·92, 1·26)

Outcomes by 2 hour post-load glucose category[a] and by 1SD				
**Pre-eclampsia**				
1 (Reference)	1·00	-	1·00	-
2	0·87	(0·49, 1·53)	0·75	(0·42, 1·34)
3	0·97	(0·55, 1·71)	0·79	(0·44, 1·43)
4	1·15	(0·57, 2·35)	0·77	(0·36, 1·62)
5	1·10	(0·52, 2·33)	0·78	(0·35, 1·71)
6	0·93	(0·27, 3·16)	0·64	(0·18, 2·25)
7	2·08	(0·70, 6·19)	1·63	(0·53, 4·99)
Per 1 SD	1·13	(0·92, 1·38)	1·00	(0·81, 1·24)
**Preterm delivery**				
1 (Reference)	1·00	-	1·00	-
2	1·07	(0·70, 1·62)	1·05	(0·69, 1·60)
3	1·03	(0·67, 1·58)	1·01	(0·65, 1·56)
4	1·19	(0·68, 2·07)	1·17	(0·67, 2·06)
5	0·89	(0·48, 1·64)	0·91	(0·49, 1·67)
6	1·61	(0·76, 3·42)	1·64	(0·76, 3·53)
7	-			
Per 1 SD	1·00	(0·86, 1·15)	1·00	(0·86, 1·17)
**Shoulder dystocia[b]**				
1 (Reference)	1·00	-	1·00	-
2	1·24	(0·40, 3·80)	1·12	(0·36, 3·46)
3	2·39	(0·86, 6·68)	2·29	(0·79, 6·65)
4	2·07	(0·55, 7·75)	2·01	(0·50, 8·04)
5	3·53	(1·11, 11·22)	3·45	(1·04, 11·51)
6	3·13	(0·60, 16·36)	3·05	(0·59, 15·73)
7	5·23	(0·99, 27·58)	6·18	(1·09, 34·96)
Per 1 SD	1·58	(1·14, 2·20)	1·63	(1·14, 2·34)
**Instrumental vaginal delivery[b]**				
1 (Reference)	1·00	-	1·00	-
2	0·81	(0·58, 1.12)	0·79	(0·56, 1·12)
3	1·02	(0·74, 1.40)	1·08	(0·76, 1·52)
4	1·32	(0·87, 2.00)	1·56	(0·99, 2·47)
5	1·05	(0·68, 1.62)	1·07	(0·67, 1·73)
6	1·37	(0·73, 2.57)	1·82	(0·94, 3·52)
7	1·81	(0·88, 3.71)	1·93	(0·87, 4·26)
Per 1 SD	1·10	(0·98, 1.24)	1·16	(1·02, 1·32)
**Intensive neonatal care**				
1 (Reference)	1·00	-	1·00	-
2	0·79	(0·52, 1·19)	0·77	(0·50, 1·17)
3	0·78	(0·51, 1·19)	0·76	(0·50, 1·16)
4	0·90	(0·51, 1·57)	0·88	(0·50, 1·54)
5	0·80	(0·45, 1·44)	0·79	(0·44, 1·41)
6	0·43	(0·13, 1·41)	0·43	(0·13, 1·42)
7	1·27	(0·49, 3·30)	1·28	(0·49, 3·36)
Per 1 SD	0·99	(0·85, 1·15)	0·98	(0·85, 1·15)

Models adjusted for gestational age at OGTT, presence or absence of family history of diabetes, previous GDM, previous macrosomia, smoking status, mother's age and mother's BMI, mothers education, baby sex and parity. Birth weight>90th percentile, sum of skinfolds >90th percentile and

preterm delivery additionally adjusted for squared BMI. Shoulder dystocia models not adjusted for previous GDM due to small numbers.

[a]Glucose categories are defined as follows: fasting plasma glucose level – category 1, <4·3 mmol/L; category 2, 4·3 – 4·4 mmol/L; category 3, 4·5 – 4·7 mmol/L; category 4, 4·8 – 4·9 mmol/L; category 5, 5·0 – 5·2 mmol/L; category 6, 5·3 – 5·6 mmol/L; category 7, 5·7 – 6·0 mmol/L. Post-load plasma glucose level – category 1, <4·7 mmol/L; category 2, 4·7 – 5·4 mmol/L; category 3, 5·5 – 6·2 mmol/L; category 4, 6·3 – 6·6 mmol/L; category 5, 6·7 – 7·2 mmol/L; category 6, 7·3 – 7·5 mmol/L; category 7, 7·6 – 7·7 mmol/L.

[b]These analyses exclude women who had a Caesarean section, therefore the N=3420

Figure S1: Frequency of primary outcomes across glucose categories using data from all pregnancies (N=9509)

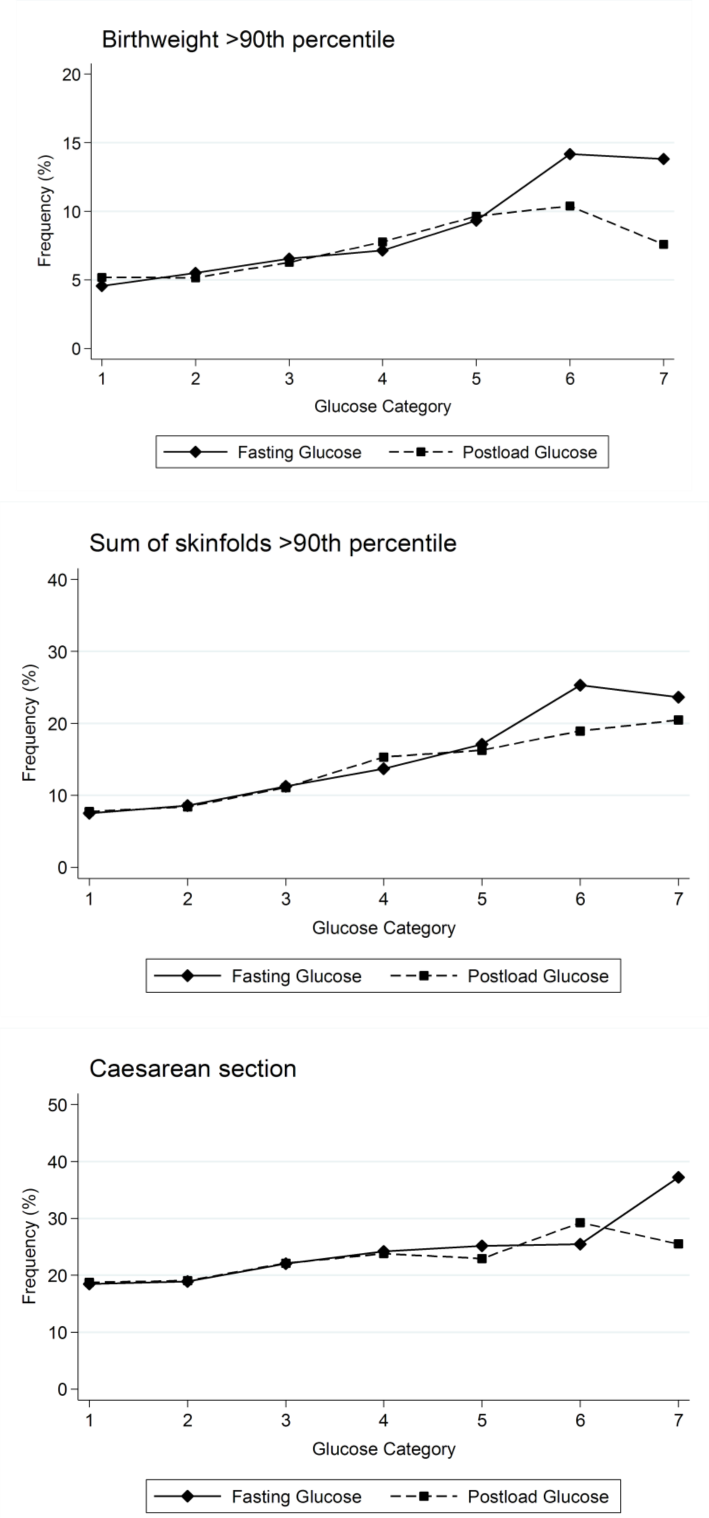

Glucose categories are defined as follows: fasting plasma glucose level – category 1, <4·3 mmol/L; category 2, 4·3 – 4·4 mmol/L; category 3, 4·5 – 4·7 mmol/L; category 4, 4·8 – 4·9 mmol/L; category 5, 5·0 – 5·2 mmol/L; category 6, 5·3 – 5·6 mmol/L; category 7, 5·7 – 6·0 mmol/L. Post-load plasma glucose level – category 1, <4·7 mmol/L; category 2, 4·7 – 5·4 mmol/L; category 3, 5·5 – 6·2 mmol/L; category 4, 6·3 – 6·6 mmol/L; category 5, 6·7 – 7·2 mmol/L; category 6, 7·3 – 7·5 mmol/L; category 7, 7·6 – 7·7 mmol/L.

Figure S2: Frequency of secondary outcomes across glucose categories by ethnicity (White British (N=3888) and South Asian (N=4821).

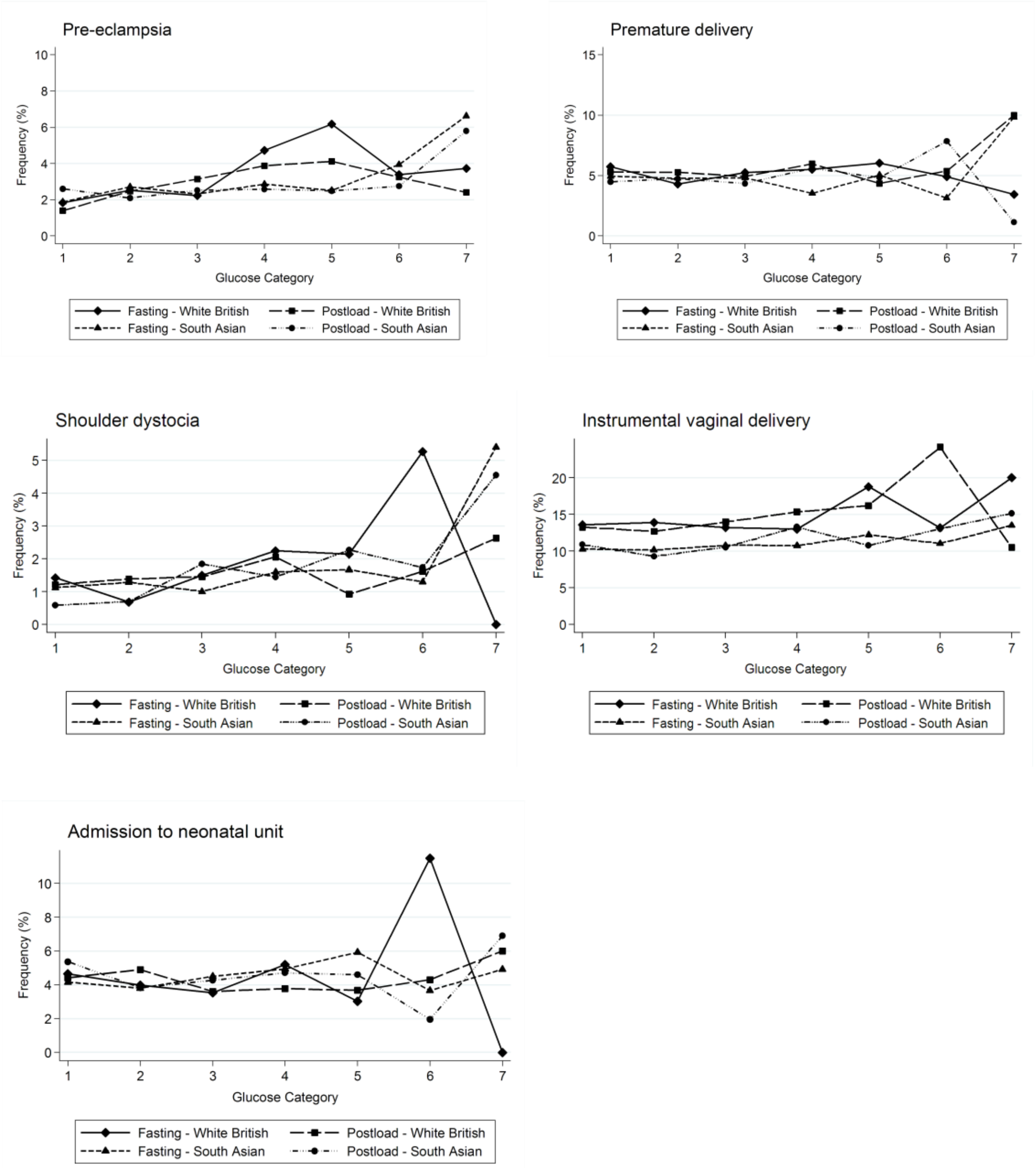

Glucose categories are defined as follows: fasting plasma glucose level – category 1, <4·3 mmol/L; category 2, 4·3 – 4·4 mmol/L; category 3, 4·5 – 4·7 mmol/L; category 4, 4·8 – 4·9 mmol/L; category 5, 5·0 – 5·2 mmol/L; category 6, 5·3 – 5·6 mmol/L; category 7, 5·7 – 6·0 mmol/L. Post-load plasma glucose level – category 1, <4·7 mmol/L; category 2, 4·7 – 5·4 mmol/L; category 3, 5·5 – 6·2 mmol/L; category 4, 6·3 – 6·6 mmol/L; category 5, 6·7 – 7·2 mmol/L; category 6, 7·3 – 7·5 mmol/L; category 7, 7·6 – 7·7 mmol/L.

For plots of shoulder dystocia and instrumental vaginal delivery women who had a Caesarean section are excluded, therefore N = 3018 for White British and 3914 for South Asian.

Figure S3: Frequency of secondary outcomes across glucose categories for all pregnancies (N = 9509). Vaginal births only for shoulder dystocia and instrumental delivery

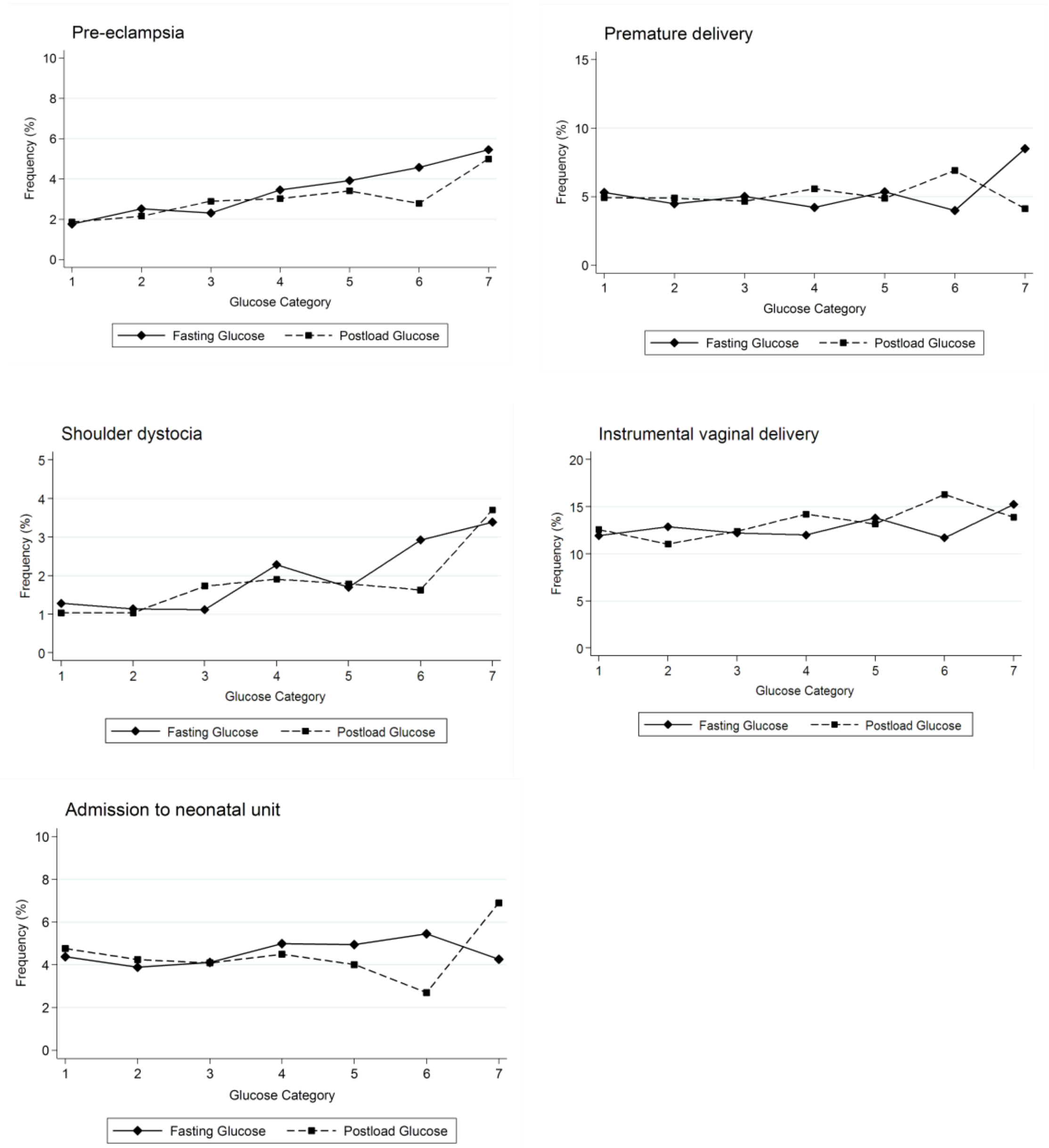

Glucose categories are defined as follows: fasting plasma glucose level – category 1, <4·3 mmol/L; category 2, 4·3 – 4·4 mmol/L; category 3, 4·5 – 4·7 mmol/L; category 4, 4·8 – 4·9 mmol/L; category 5, 5·0 – 5·2 mmol/L; category 6, 5·3 – 5·6 mmol/L; category 7, 5·7 – 6·0 mmol/L. Post-load plasma glucose level – category 1, <4·7 mmol/L; category 2, 4·7 – 5·4 mmol/L; category 3, 5·5 – 6·2 mmol/L; category 4, 6·3 – 6·6 mmol/L; category 5, 6·7 – 7·2 mmol/L; category 6, 7·3 – 7·5 mmol/L; category 7, 7·6 – 7·7 mmol/L.
For plots of shoulder dystocia and instrumental delivery women who had a Caesarean section are excluded, therefore N = 7526.

References of included studies from the systematic review reported in the ‘Research in context’ section of the main paper

Aris IM, Soh SE, Tint MT, Liang S, Chinnadurai A, Saw SM, et al. Effect of Maternal Glycemia on Neonatal Adiposity in a Multiethnic Asian Birth Cohort. J Clin Endocrinol Metab. 2014; 99(1): 240–7.

Carr DB, Newton KM, Utzschneider KM, Faulenbach MV, Kahn SE, Easterling TR, et al. Gestational diabetes or lesser degrees of glucose intolerance and risk of preeclampsia. Hypertens Pregnancy. 2011; 30(2): 153-63.

Chandna A, Zuberi LM, Munim S. Threshold values for the glucose challenge test in pregnancy. Int J Gynaecol Obstet. 2006; 94(2): 119-20.

Cheng YW, McLaughlin GB, Esakoff TF, Block-Kurbisch I, Caughey AB. Glucose challenge test: screening threshold for gestational diabetes mellitus and associated outcomes. J Mat-fetal & neonatal med 2007; 20(12): 903-8.

Dunne F. Atlantic Diabetes in Pregnancy cohort. Personal communication. 2014.

Figueroa D, Landon MB, Mele L, Spong CY, Ramin SM, Casey B, et al. Relationship between 1-hour glucose challenge test results and perinatal outcomes. Obstet Gynecol. 2013; 121(6): 1241-7.

HAPO Study Cooperative Research Group. Hyperglycemia and adverse pregnancy outcome (HAPO) study: preeclampsia. Am J Obstet Gynecol. 2010; 202(3): e1-e7.

Hillier TA, Pedula KLMS, Vesco KK, Schmidt MM, Mullen JA, LeBlanc ES, et al. Excess Gestational Weight Gain: Modifying Fetal Macrosomia Risk Associated With Maternal Glucose. Obstet Gynecol. 2008; 112(5): 1007-14.

Jensen DM, Damm P, Sorensen B, Molsted-Pedersen L, Westergaard JG, Klebe J, et al. Clinical impact of mild carbohydrate intolerance in pregnancy: a study of 2904 nondiabetic Danish women with risk factors for gestational diabetes mellitus. Am J Obstet Gynecol. 2001; 185(2): 413-9.

Kerényi Z, Tamás G, Kivimäki M, Péterfalvi A, Madarász E, Bosnyák Z, et al. Maternal Glycemia and Risk of Large-for-Gestational-Age Babies in a Population-Based Screening. Diabetes Care. 2009; 32(12): 2200-5.

Landon MB, Mele L, Spong CY, Carpenter MW, Ramin SM, Casey B, et al. The Relationship Between Maternal Glycemia and Perinatal Outcome. Obstet Gynecol. 2011; 117(2, Part 1): 218-24.

Lao TT, Ho LF. Does maternal glucose intolerance affect the length of gestation in singleton pregnancies? J Soc Gynecol Invest. 2003; 10(6): 366-71.

Little RR, McKenzie EM, Shyken JM, Winkelmann SE, Ramsey LM, Madsen RW, et al. Lack of Relationship Between Glucose Tolerance and Complications of Pregnancy in Nondiabetic Women. Diabetes Care. 1990; 13(5): 483-7.

Lurie R, Levy R, Weiss G, Boutin ZJ, Hagay S. Low values on 50 gram glucose challenge test or oral 100 gram glucose tolerance test are associated with good perinatal outcome. J Obstet Gyaecol. 1998; 18(5): 451-4.

Metzger BE, Lowe LP, Dyer AR, et al, and the HAPO Study Cooperative Research Group. hyperglycemia and adverse pregnancy outcomes. N Engl J Med 2008; 358: 1991–2002.

Metzger BE, Persson B, Lowe LP, Dyer AR, Cruickshank JK, Deerochanawong C, et al. Hyperglycemia and Adverse Pregnancy Outcome Study: Neonatal Glycemia. Pediatrics. 2010; 126(6): e1545-e52.

Moses RGF, Calvert DP. Pregnancy Outcomes in Women Without Gestational Diabetes Mellitus Related to the Maternal Glucose Level: Is there a continuum of risk? Diabetes Care. 1995; 18(12): 1527-33.

Ong KK, Diderholm B, Salzano G, Wingate D, Hughes IA, MacDougall J, et al. Pregnancy insulin, glucose, and BMI contribute to birth outcomes in nondiabetic mothers. Diabetes Care. 2008; 31(11): 2193-8.

Pettitt DJ, Knowler WC, Baird HR, Bennett PH. Gestational diabetes: infant and maternal complications of pregnancy in relation to third-trimester glucose tolerance in the Pima Indians. Diabetes Care. 1980; 3(3): 458-64.

Riskin-Mashiah S, Younes G, Damti A, Auslender R. First-trimester fasting hyperglycemia and adverse pregnancy outcomes. Diabetes Care. 2009; 32(9): 1639-44.

Savona-Ventura C, Craus J, Vella K, Grima S. Lowest threshold values for the 75g oral glucose tolerance test in pregnancy. Malta Med J. 2010; 22(1): 18-20.

Scholl TO, Sowers M, Chen X, Lenders C. Maternal glucose concentration influences fetal growth, gestation, and pregnancy complications. American Journal of Epidemiology. 2001; 6: 514-20.

Sermer M, Naylor CD, Gare DJ, Kenshole AB, Ritchie JWK, Farine D, et al. Impact of increasing carbohydrate intolerance on maternal-fetal outcomes in 3637 women without gestational diabetes: The Toronto tri-hospital gestational diabetes project. Am J Obstet Gynecol. 1995; 173: 146-56.

Subramaniam A, Jauk V, Tita A, Harper L. Interaction between maternal obesity and 1-hour glucose challenge test results on maternal and perinatal outcomes. Am J Obs Gynecol. 2014; 210 (1): S132.

Tallarigo L, Giampietro O, Penno G, Miccoli R, Gregori G, Navalesi R. Relation of Glucose Tolerance to Complications of Pregnancy in Nondiabetic Women. N Engl J Med. 1986; 315(16): 989-92.

Witter F, Niebyl J. Abnormal glucose screening in pregnancy in patients with normal oral glucose tolerance tests as a screening test for fetal macrosomia. Int J Gynaecol Obstet. 1988; 27(2): 181-4.

Wright J. Born in Bradford cohort. Personal communication. 2014.

Yee LM, Cheng YW, Liddell J, Block-kurbisch I, Caughey AB. 50-Gram glucose challenge test: is it indicative of outcomes in women without gestational diabetes mellitus? J Maternal-Fetal Neonatal Med. 2011; 24(9): 1102-6.
